# Diagnostic classifications based on cognitive, CSF, and PET biomarkers across ADNI cohorts: cross-sectional and longitudinal variability

**DOI:** 10.1093/braincomms/fcag312

**Published:** 2026-08-26

**Authors:** Maryam Fotouhi, Helena C Chui, Jeiran Choupan, Neda Jahanshad, Steven Cen, Bino A Varghese, John Matthew Ringman, Lon S Schneider, Nasim Sheikh-Bahaei

**Affiliations:** Laboratory of Neuroimaging, USC Mark and Mary Stevens Neuroimaging and Informatics Institute, Keck School of Medicine, University of Southern California, Los Angeles, CA 90033, USA; Alzheimer's Disease Research Center, Keck School of Medicine, University of Southern California, Los Angeles, CA 90033, USA; Department of Neurology, Keck School of Medicine, University of Southern California, Los Angeles, CA 90033, USA; Laboratory of Neuroimaging, USC Mark and Mary Stevens Neuroimaging and Informatics Institute, Keck School of Medicine, University of Southern California, Los Angeles, CA 90033, USA; NeuroScope Inc., Scarsdale, NY, USA; Department of Neurology, Keck School of Medicine, University of Southern California, Los Angeles, CA 90033, USA; USC Mark and Mary Stevens Neuroimaging and Informatics Institute, Keck School of Medicine of USC, Marina del Rey, CA 90292, USA; Department of Neurology, Keck School of Medicine, University of Southern California, Los Angeles, CA 90033, USA; Department of Radiology, Keck School of Medicine, University of Southern California, Los Angeles, CA 90033, USA; Department of Radiology, Keck School of Medicine, University of Southern California, Los Angeles, CA 90033, USA; Department of Neurology, Keck School of Medicine, University of Southern California, Los Angeles, CA 90033, USA; Alzheimer's Disease Research Center, Keck School of Medicine, University of Southern California, Los Angeles, CA 90033, USA; Department of Neurology, Keck School of Medicine, University of Southern California, Los Angeles, CA 90033, USA; Department of Psychiatry and Behavioral Sciences, University of Southern California, Los Angeles, CA 90033, USA; Department of Radiology, Keck School of Medicine, University of Southern California, Los Angeles, CA 90033, USA

**Keywords:** Alzheimer’s disease, biomarkers, cognitive trajectories, heterogeneity, disease progression

## Abstract

Alzheimer’s disease and related neurodegenerative disorders exhibit marked clinicobiological heterogeneity, posing challenges for accurate diagnosis, prognosis and therapeutic development. Using data from multiple cohorts of the Alzheimer’s Disease Neuroimaging Initiative (ADNI), we conducted comprehensive cross-sectional and longitudinal concordance analyses of dementia severity, CSF and PET amyloid and tau. We also examined the stability of biomarker positivity and cognitive status (stable, progressive, reversive and fluctuating) and their inter-relationships. The ability to separate methodological from biological sources of variability is relevant to understanding the robustness of the Amyloid/Tau/Neurodegeneration classification framework, which is particularly important in the era of disease-modifying therapies. Our data revealed both temporal and spatial discrepancies among variables used for classifying the level of cognitive impairment and between the methods used for classifying positive amyloid or tau status (CSF/PET). Methodological discrepancies in region-specific definitions, positivity thresholds and segmentation constraints contributed to PET inconsistencies. Additionally, biomarker status for CSF and PET positivity fluctuated over time, and there was no evidence of temporal precedence for either CSF or PET. There was pronounced spatiotemporal heterogeneity in tau-PET patterns, with implications for the clinical trajectory. Initial tau deposition in the entorhinal cortex, compared to non-limbic meta-temporal regions, was associated with more rapid cognitive decline and greater amyloid burden. Notably, a subset of mild cognitive impairment cases demonstrated diagnostic reversion to cognitively normal status and showed lower amyloid and tau burden and a higher prevalence of affective symptoms, possibly representing individuals without or at an early stage of Alzheimer’s pathology. Results underscore the complexity and variability of Alzheimer’s disease phenotypes and progression and provide a sanguine perspective on current diagnostic frameworks. Recognizing variability sources in biomarkers is particularly important when diagnosis relies on a single biomarker or cross-sectional data. More importantly, given the complex interplay of factors contributing to dementia and cognitive decline, deep learning and artificial intelligence-based algorithms are needed for precise and individualized diagnoses. Harmonized and standardized datasets with clearly defined variables, without excluding atypical cases, are needed for the effectiveness of such machine learning models. Therefore, investigating Alzheimer’s disease biomarker heterogeneity is critical towards maximizing the potential of artificial intelligence in integrating multi-dimensional clinical and biomarker data beyond human-centred interpretation. In this comprehensive work, we provide insights into the observed heterogeneities and their potential contributing factors. We aim to use this insight and continue this effort by harmonizing the conflicting data, making them suitable for diagnostic algorithms and sharing the harmonized dataset in the near future.

## Teaser

Fotouhi *et al*. report marked heterogeneity in Alzheimer’s disease progression, demonstrating discordance between CSF and PET biomarker positivity and mismatches between clinical diagnosis and cognitive trajectories. These results underscore the importance of accounting for biomarker and clinical variability when interpreting disease status in the era of anti-amyloid therapy.

## Graphical Abstract

**Graphical Abstract:**
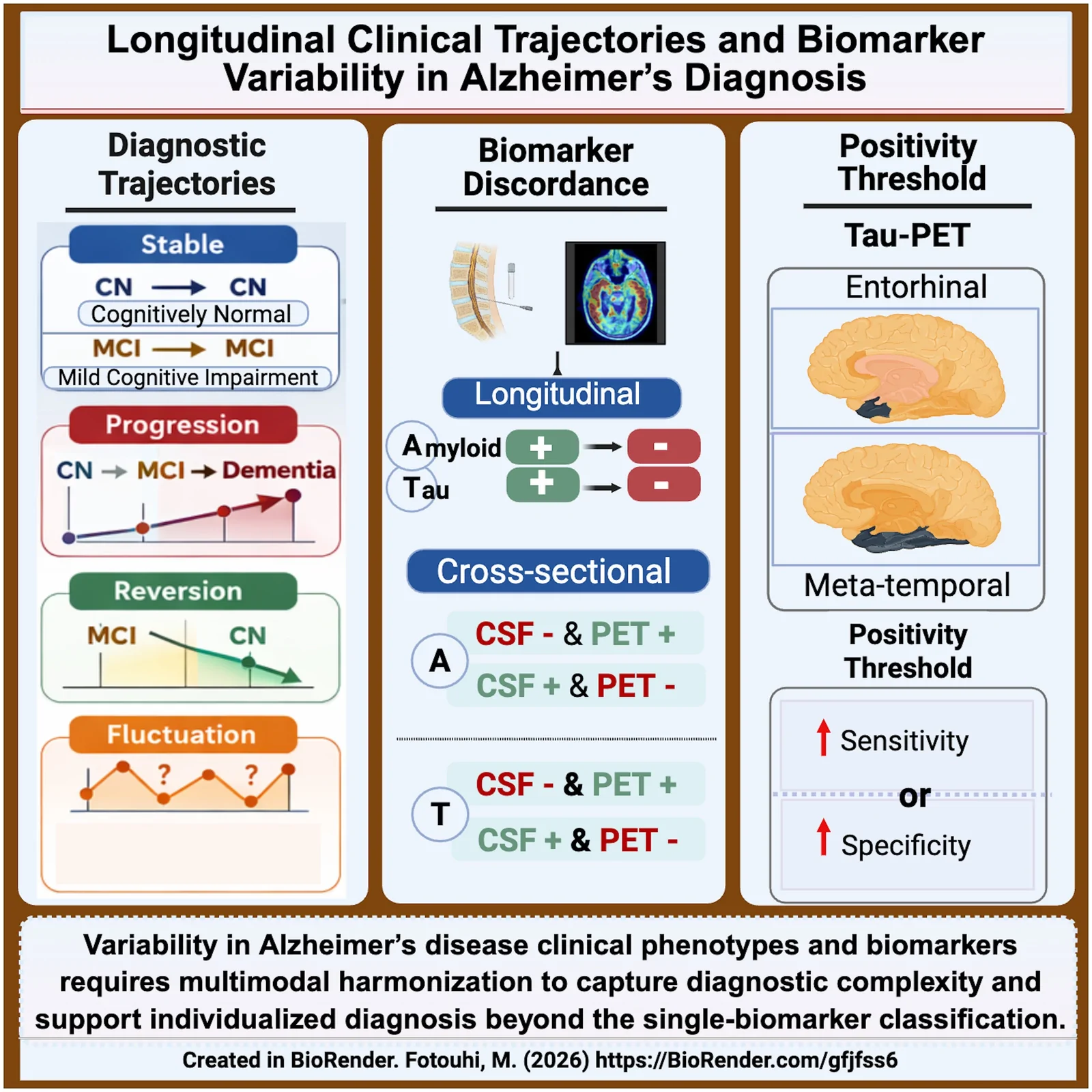

## Introduction

Neurodegenerative disorders, including Alzheimer's disease, exhibit biological heterogeneity, resulting in significant variability within *in vivo* disease biomarkers. Accurate diagnosis of Alzheimer's disease dementia and mild cognitive impairment (MCI) is paramount for the timely initiation of treatment and for predicting the efficacy of disease-modifying therapies. Historically, the diagnosis of Alzheimer's disease has relied on clinical presentations and cognitive assessments, taking into account the comprehensive patient profile and ruling out differential diagnoses.^1,2^ Over the past two decades, research in Alzheimer's disease has led to the development of more accurate biomarker-based diagnostic and staging tools. These advancements integrate CSF measurements, along with PET and MRI, and, more recently, plasma markers, often using the Amyloid/Tau/Neurodegeneration categorization.^3^ Although the introduction of additional biomarkers and surrogate markers has revolutionized the field and significantly improved the diagnostic accuracy of Alzheimer's disease, the complexity of integrating clinical findings, genetic factors, cognitive scores and multiple biomarker measures often exceeds the capacity for consistent human interpretation. Years of studying these complex pathologies have taught us that simplifying the disease process or overlooking some of the contributing factors is not an effective strategy for understanding the pathophysiology or predicting outcomes. Therefore, there is a growing need for artificial intelligence (AI) and machine learning (ML) models that enable integration of different factors without excluding atypical cases. This approach can refine disease staging, enhance prognostic accuracy and ultimately improve patient selection and outcome prediction in clinical trials.

More importantly, extensive data indicate that Alzheimer's disease course and clinical presentations can undergo significant fluctuations. These fluctuations may also manifest in the data extracted from biomarkers, including the volumetric measurements from imaging, the burden of abnormal proteins in PET or protein levels in the CSF. This can lead to substantial uncertainty and contradictory results in research and the clinical management of these patients.^4,5^

In addition, heterogeneity across cohorts and studies has constrained the utility of large-scale longitudinal cohorts in Alzheimer's disease research. Harmonizing diagnostic criteria, aligning biomarker profiles and redefining disease categories through integrated clinical and molecular indicators can mitigate this heterogeneity, thereby broadening eligibility to include atypical phenotypes. Artificial intelligence and deep learning methodologies offer considerable potential for improving diagnostic accuracy by integrating complex multi-dimensional data; however, their effectiveness is limited by variability in data acquisition, terminology and diagnostic standards across research settings. Notably, predictive models that incorporate heterogeneity-aware algorithms demonstrate up to a 10% improvement in area under the curve compared with conventional models.^6-8^

This study tackles major challenges in employing extensive Alzheimer’s disease datasets for clinical research and enhancing the quality of retrospective data. Using the complete Alzheimer’s Disease Neuroimaging Initiative (ADNI) dataset, we systematically examined diagnostic discrepancies, longitudinal biomarker trajectories and the variability in disease progression and reversion. Our approach includes identifying and exploring underlying methodological and clinical contributors to these inconsistencies and with the aim of recategorizing cases into clinically and biologically meaningful subgroups when appropriate in future work. By identifying these sources of variability, we intend to help standardize terminology and data, which are both important steps in making reliable machine learning models and moving forward with precision medicine in Alzheimer’s disease. The harmonized dataset produced will be made publicly available to the research community to enable effective patient stratification in therapeutic trials, thereby improving the clinical relevance and translational significance of Alzheimer's disease research.

## Materials and methods

### Participants and multi-modal assessments

Participants for the present study were drawn from all stages of the ADNI, including ADNI 1, ADNI 2, ADNI GO and ADNI 3, which were included after screening (Table 1). The ADNI includes individuals aged 55 to 90 (http://adni.loni.usc.edu/) and contains clinical data for subjects, such as demographic details, vital signs, cognitive evaluations, extensive medical history and imaging. We utilized CSF measurements in conjunction with amyloid and tau PET data from ADNI subjects. CSF and PET measurements, which were within 1 year of each other, were considered as the same time point and incorporated with the prior syndromic diagnosis of cognitively normal (CN), MCI or dementia to enhance the temporal correlation between PET and CSF data. Clinical assessments, including Mini-Mental State Examination (MMSE), Global Clinical Dementia Rating (CDR), CDR sum of boxes (CDR-SB) and Alzheimer's Disease Assessment Scale-Cognitive 13 (ADAS-Cog 13), were all obtained through clinician-administered evaluations (Fig. 1). Cognitive and clinical assessments (MMSE, CDR-SB, ADAS-Cog 13) were obtained from ADNI manuals across phases. While operational details may vary by phase, scoring outputs matched in different ADNI phases and were analysed using the same derived variables across all phases. Information on past psychiatric symptom history and medication use was collected through patient or caregiver reports. The ADNI criteria for cognitively normal, MCI and dementia syndrome are listed in the Supplementary Methods.

**Figure 1:**
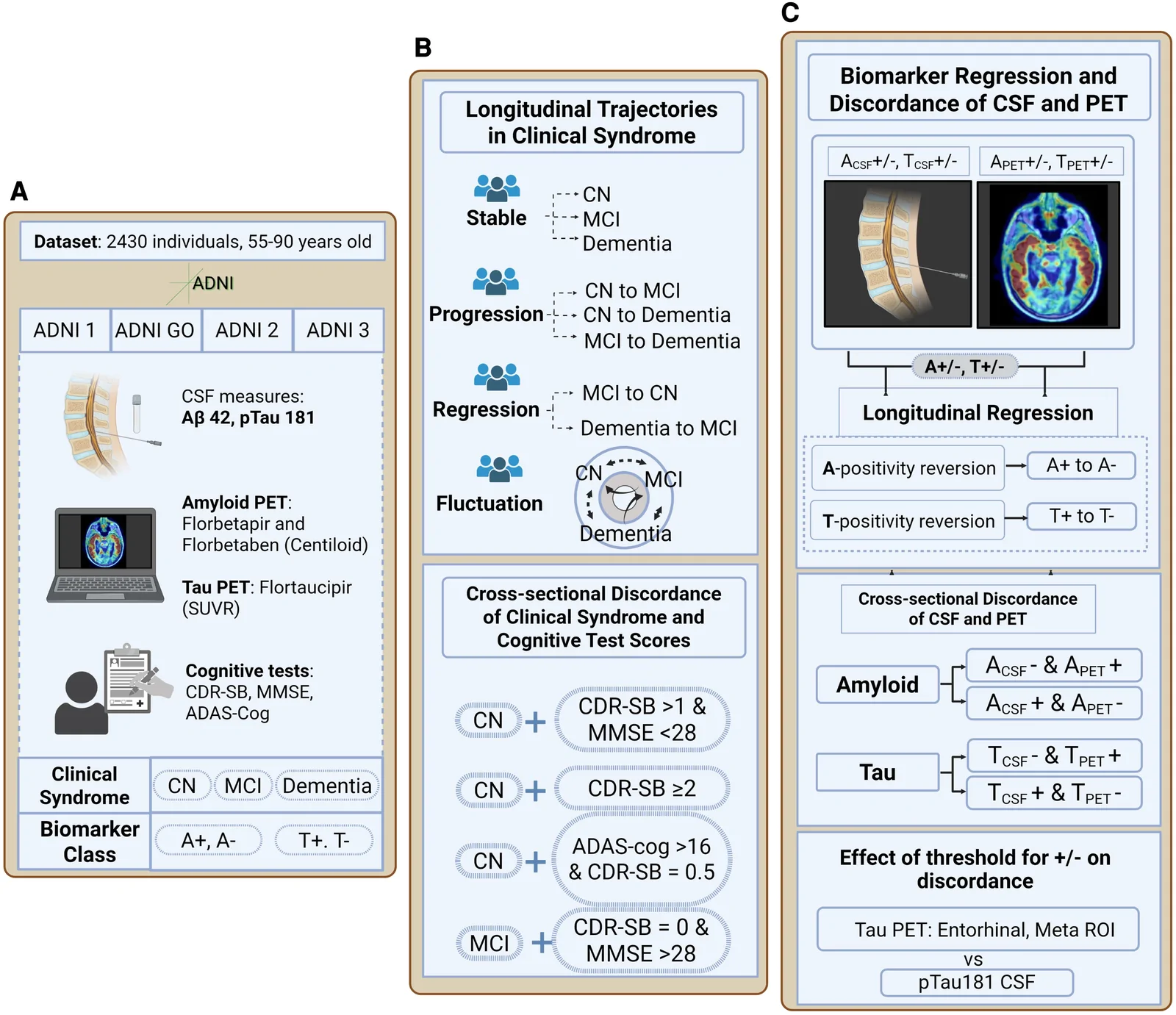
**Overview of cohort selection, clinical trajectory classification and biomarker discordance analysis.** (**A**) Participants from all phases of the Alzheimer’s Disease Neuroimaging Initiative (ADNI), including ADNI 1, GO, 2 and 3, were included to evaluate heterogeneity across syndromic diagnosis, cognitive measures and CSF and PET biomarkers. Clinical and biomarker variables included syndromal diagnosis [cognitively normal (CN), mild cognitive impairment (MCI) and dementia], cognitive assessments, CSF amyloid-β42 (Aβ42), CSF phosphorylated tau 181 (p-tau181), amyloid PET centiloid values, tau PET standardized uptake value ratio (SUVR) measures in entorhinal and meta-temporal and amyloid and tau positivity or negativity classifications. (**B**) Longitudinal clinical trajectories were classified as stable, progressive, reversive or fluctuating based on changes in syndromal diagnosis over follow-up visits. Cross-sectional diagnostic discrepancies were assessed by matching clinical diagnostic labels with cognitive test performance, including the Clinical Dementia Rating Sum of Boxes (CDR-SB), Mini-Mental State Examination (MMSE) and Alzheimer’s Disease Assessment Scale-Cognitive Subscale 13-item version (ADAS-Cog13). (**C**) Biomarker discordance was assessed longitudinally for changes in amyloid and tau positivity and cross-sectionally for disagreement between CSF/PET amyloid or tau positivity. Tau PET discordance was further evaluated across entorhinal and meta-temporal regions and at different thresholds, with higher sensitivity or specificity in diagnostic classification for Alzheimer’s disease pathology. Created in BioRender. Fotouhi, M. (2026) https://BioRender.com/2s9h6np.

**Table 1:** Demographic, clinical, cognitive, and biomarker characteristics across ADNI cohorts

Variable	Total	ADNI 1	ADNI 2	ADNI GO	ADNI 3	*P-value* *
Number of individuals	2430	819	790	131	690	-
Baseline age, years	72.9 ± 7.4	75.2 ± 6.8	72.7 ± 7.2	71.5 ± 7.9	70.7 ± 7.4	<0.0001
Follow up, years	4.1 ± 3.6	4.8 ± 4.2	4.7 ± 3.5	5.9 ± 3.5	2.2 ± 1.7	<0.0001
Sex, F	1157. 47.6%	342, 41.8%	379, 48%	60, 45.8%	376, 54.5%	<0.0001
APOE e4 carrier	1014, 45.8%	400, 48.8%	358, 46%	52, 40.6%	204, 41.9%	0.059
ADAS-Cog 13	15.8 [15.5–16.2] (2405)	18.4 [17.8–19.1] (811)	16.1 [15.4–16.8] (783)	12.40 [11.5–13.3] (130)	13.1 [12.5–13.8] (681)	<0.0001
MMSE	27.4 [27.3–27.5] (2429)	26.7 [26.6–27] (819)	27.4 [27.2–27.6] (790)	28.3 [28–28.6] (131)	27.9 [27.7–28.1] (689)	<0.0001
CDR	0.36 [0.35–0.37] (2430)	0.42 [0.40–0.44] (819)	0.37 [0.35–0.39] (790)	0.50 [0.50–0.50] (131)	0.26 [0.23–0.28] (690)	<0.0001
CDR-SB	1.46 [1.39–1.53] (2430)	1.80 [1.67–1.92] (819)	1.54 [1.42–1.67] (790)	1.24 [1.12–1.36] (131)	1.02 [0.89–1.14] (690)	<0.0001
CSF AB-42	1084.9 [1054.6–1115.2] (1660)	908.2 [856.2–960.2] (431)	1104.7 [1059–1150.4] (711)	1267.2 [1147–1387.4] (118)	1186.4 [1119.8–1253] (400)	<0.0001
CSF pTAU-181	26.7 [26–27.4] (1650)	29.6 [28.2–31] (426)	26.9 [25.9–28] (710)	24.5 [22.1–27] (118)	23.7 [22.4–25] (396)	<0.0001
Amyloid, PET Centiloid	36 [33.8–38.2] (1724)	34.8 [28.2–41.5] (204)	41.1 [37.7–44.5] (769)	31.4 [23.8–39] (129)	31.1 [23.8–39] (622)	0.0004
Entorhinal Tau-PET SUVR	1.26 [1.24–1.28] (931)	1.28 [1.22–1.30] (54)	1.26 [1.22–1.30] (234)	1.25 [1.16–1.28] (37)	1.26 [1.24–1.28] (606)	0.9
Meta-temporal Tau-PET SUVR	1.29 [1.28–1.31] (931)	1.29 [1.23–1.34] (54)	1.30 [1.26–1.34] (234)	1.28 [1.20–1.40] (37)	1.29 [1.27–1.32] (606)	0.9

Baseline age and follow-up duration = years (mean ± SD); Sex (F) = number of females (%); APOE ε4 carrier = number of carriers (%); ADAS-Cog 13 = Alzheimer’s Disease Assessment Scale-Cognitive Subscale (13-item), mean (95% CI); MMSE = Mini-Mental State Examination, mean (95% CI); CDR = Clinical Dementia Rating, mean (95% CI); CDR-SB = CDR Sum of Boxes, mean (95% CI); CSF Aβ42 = cerebrospinal fluid amyloid-β42, mean (95% CI); CSF p-tau181 = cerebrospinal fluid phosphorylated tau-181, mean (95% CI); Amyloid PET Centiloid = global cortical amyloid burden, mean (95% CI); Entorhinal Tau-PET SUVR = tau standardized uptake value ratio in the entorhinal cortex, mean (95% CI); Meta-Temporal Tau-PET SUVR = tau standardized uptake value ratio in the meta-temporal region, mean (95% CI); ADNI-1, ADNI-GO, ADNI-2, ADNI-3 = phases of the Alzheimer’s Disease Neuroimaging Initiative.

Cognitive, CSF and PET measures reported in this table are based on the first available data for each case. If not all participants had data available for a given measure, the number of available cases is shown in parentheses within the corresponding cell.

^*^ *P* values represent comparisons across ADNI cohorts. Continuous variables were compared using one-way ANOVA and Tukey’s Honestly Significant Difference (HSD) *post hoc* tests. Categorical variables were compared using chi-square tests, followed by Bonferroni-corrected pairwise comparisons. Detailed *post hoc* results are provided in the Supplementary Tables.

### Assessing inhomogeneity and variability

#### Syndromic diagnosis

The ADNI cohort's clinical diagnostic labels were used to group participants into three groups: CN, MCI or dementia. These classifications are based on the original ADNI clinical consensus diagnoses and were used exactly as they were, without any changes for this study (Table 1).

#### Further classification based on longitudinal trajectory of cognitive impairment

Individuals were classified into four groups: stable, progressive, reversive and fluctuating based on the level of cognitive impairment (CN, MCI, dementia) assessed over time. A minimum follow-up duration of ≥ 2 years was implemented for trajectory classification. Participants were considered stable if their syndromal classification remained the same at baseline throughout the follow-up periods ranging from 3 months to 17 years. Individuals who progressed from CN to MCI, MCI to dementia or CN to dementia were classified as progressive. Participants who transitioned to a less severe level of cognitive impairment (e.g. from MCI to CN or from dementia to MCI) were considered reversives. Individuals were classified as fluctuating when there was variation in the longitudinal course, exhibiting both improvement (reversion) and deterioration (progression) (Table 2).

**Table 2:** Trajectories in clinical diagnosis across ADNI participants with at least two years follow-up

Clinical diagnosis	Stable, *N* = 1016			Progression, *N* = 431			Reversion, *N* = 60			*P*-value*
Trajectory	CN	MCI	Dementia	CN to MCI	CN to dementia	MCI to dementia	MCI to CN	Dementia to MCI	Fluctuation	
Number of individuals	501	347	168	82	33	316	57	3	80	-
Baseline Age, years	72.4 ± 6.2	72.7 ± 7.5	75.6 ± 7.8	75.4 ± 5.9	75.2 ± 4	74 ± 7	68.2 ± 6.8	73; 76; 82	71.6 ± 7.7	<0.0001
Follow-up, years	5.5 ± 3.2	4.9 ± 2.8	2.9 ± 1.4	6.6 ± 3.6	9.2 ± 3.7	5.4 ± 2.8	6.8 ± 3.7	2; 3; 4	8 ± 3.6	<0.0001
Sex (F), *N*, %	291, 58.1%	141, 40.6%	71, 42.3%	42, 51.2%	17, 51.5%	129, 40.8%	28, 49.1%	0	27, 33.8%	<0.0001
APOE e4 carrier, *N*, %	148, 29.7%	128, 37.4%	108, 65.5%	29, 35.8%	14, 42.4%	201, 64.4%	21, 37.5%	No; Yes; No	32, 40.5%	<0.0001
Baseline ADAS-cog 13	8.1 [7.7–8.4]	13.9 [13.3–14.4]	29.2 [28.1–30.3]	11 [10–12]	10.2 [8.3–12]	20.4 [19.7–21]	9.2 [8.2, 10.2]	20.6; 19.6; 13.4	12.4 [11–13.8]	<0.0001
Baseline MMSE	29.1 [29–29.2]	28 [27.8–28.2]	23 [22.7–23.3]	28.9 [28.6–29.2]	29.5 [29.2–29.7]	27 [26.8–27.2]	28.9 [28.6–29.2]	22; 26;28	28.2 [27.8–28.6]	<0.0001
Baseline CDR	0	0.5 [0.50–0.51]	0.75 [0.71–0.79]	0.01 [0–0.02]	0	0.5 [0.5–0.5]	0.5 [0.47–0.51]	1; 1; 0.5	0.4 [0.3–0.4]	<0.0001
Baseline CDR-SB	0.03 [0.02–0.04]	1.2 [1.14–1.3]	4.3 [4.1–4.6]	0.1 [0.03–0.11]	0.05 [0–0.1]	1.9 [1.8–2]	1 [0.8–1.2]	4.5; 4.5; 1.5	1.09 [0.8–1.3]	<0.0001
A+, *N*, %	159, 34.8%	153, 47.8%	114, 89.8%	37, 48.7%	21, 72.4%	224, 88.2%	22, 42.3%	A+; −; −	41, 54.7%	<0.0001
T+, *N*, %	131, 30.3%	127, 42.6%	107, 85.6%	34, 49.3%	14, 56%	176, 75.5%	11, 22.9%	T+; −; −	24, 33.8%	<0.0001
Cardiovascular disease in medical history, *N*, %	209, 65.3%	216, 73.2%	93, 65.5%	54, 78.3%	23, 71.9%	213, 72.5%	29, 63%	−; Yes; Yes	49, 65.3%	0.15
Psychiatric symptom in medical history, *N*, %	83, 25.9%	114, 38.6%	62, 43.7%	19, 27.5%	12, 37.5%	128, 43.5%	18, 39.1%	−; No; Yes	32, 42.7%	<0.0001
Key medication history										
Anti-depressant	115, 22.9%	124, 35.2%	36, 20.9%	28, 34.2%	18, 54.5%	138, 43.7%	20, 35.1%	1, 33.3%	28, 35%	<0.0001
Aricept	2, 0.4%	73, 20.7%	56, 32.6%	8, 9.8%	14, 42.4%	149, 47.2%	2, 3.5%	2, 66.7%	19, 23.7%	<0.0001
Namenda	1, 0.2%	34, 9.7%	50, 29.1%	2, 2.4%	10, 30.3%	130, 41.1%	2, 3.5%	1, 33.3%	13, 16.3%	<0.0001
Exelon	1, 0.2%	16, 4.6%	11, 6.4%	1, 1.2%	0	29, 9.2%	2, 3.5%	0	8, 10%	<0.0001
Razadyne	2, 0.4%	18, 5.1%	6, 3.5%	2, 2.4%	4, 12.1%	25, 7.9%	1, 1.8%	0	0	<0.0001
Other behavioral medication	48, 9.6%	52, 14.8%	14, 8.1%	7, 8.5%	6, 18.2%	64, 20.3%	5. 8.8%	1, 33.3%	12, 15%	0.0001
None of above	408, 81.3%	204, 58%	7, 4.1%	63, 76.8%	26, 78.8%	86, 27.2%	47, 82.5%	0	52, 65%	<0.0001

Baseline age and follow-up duration = years (mean ± SD); Sex (F) = number of females (%); APOE ε4 carrier = number of carriers (%); Baseline ADAS-Cog 13 = Alzheimer’s Disease Assessment Scale–Cognitive Subscale (13-item), mean (95% CI); Baseline MMSE = Mini-Mental State Examination score, mean (95% CI); Baseline CDR = Clinical Dementia Rating score, mean (95% CI); Baseline CDR-SB = CDR Sum of Boxes score, mean (95% CI); A+ = number of amyloid-positive participants (%); T+ = number of tau-positive participants (%); Cardiovascular disease in medical history = number of participants with a self-reported history (%); Psychiatric symptom in medical history = number of participants with a self-reported history (%).

All cognitive and biomarker measures reported in this table are based on the first available data for each case. For cardiovascular disease and psychiatric symptom history, participants were classified as positive if such a condition was reported at baseline, during follow-up or in their past medical history. If not all participants had data available for a given measure, the number of available cases is shown in parentheses within the corresponding cell.

^*^ *P* values represent comparisons across clinical trajectory groups. Continuous variables were compared using one-way ANOVA, followed by Tukey’s Honestly Significant Difference (HSD) *post hoc*. Categorical variables were compared using chi-square tests when expected cell counts were adequate; otherwise Fisher’s exact tests with Bonferroni correction were applied due to sparse cells. Detailed *post hoc* comparison results are provided in the Supplementary Tables.

#### Biomarker-based diagnosis

Amyloid (A) and tau (T) positivity were determined using previously established CSF^9,10^ and PET^11,12^ thresholds. CSF and PET measurements obtained within 1 year were treated as a single time point. Amyloid status was defined as positive (A+) if either CSF Aβ42 or amyloid PET was positive; cases were classified as negative (A−) if both were negative or if the only available measure was negative. Tau status was defined as negative (T−) if all available tau biomarkers (CSF p-tau181, tau PET in the entorhinal cortex and tau PET in the meta-temporal region) were negative; positivity (T+) required that any one of these measures were positive.

Cut-offs for positivity of Aβ42 and p-tau181^9,10^ were established based on published data by the biomarker core of ADNI as 977 and 24 pg/mL, respectively. Amyloid positivity was determined using PET data based on ADNI guidelines, which set a specific cut-off for amyloid-PET positivity at 18 Centiloid (CL) for [18]F-florbetaben (FBB) (1.08 SUVR) or 20 CL for [18]F-florbetapir (FBP) (1.11 SUVR), where a higher centiloid means more amyloid plaque burden.^11^ Since only one tracer for tau PET was used in ADNI, [18F] flortaucipir (FTP), the standardized uptake value ratio (SUVR) values were used, and the positivity threshold established in the previous study was 1.21 in the entorhinal cortex and 1.25 in the temporal meta region of interest (entorhinal, amygdala, fusiform, inferior temporal, middle temporal), with higher SUVRs showing greater tau burden.^13,14^ In order to reduce the influence of off-target binding in the dorsal cerebellum, regional SUVRs have been intensity-normalized by the inferior cerebellar grey matter reference region.^15^ Details of PET acquisition are described in previous publications^11,12,15,16^ and on the ADNI website (https://adni.loni.usc.edu/).

For tau PET, two thresholds for positivity were applied and assessed for discrepancy evaluation of positivity based on current literature: the Youden index threshold was used as a more inclusive criterion, selecting a cut-off that increases sensitivity by identifying a greater number of cases as tau PET positive, including those with lower tracer uptake. In contrast, the ‘mean plus two standard deviations’ threshold was applied as a more restrictive criterion, prioritizing specificity by setting a higher cut-off value and thereby limiting positivity to cases with markedly elevated tracer uptake.^12^ After discrepancy evaluation, the Youden index threshold was used for final positivity classification in this study.

In addition to threshold variations, tau positivity was defined based on spatial distribution, distinguishing cases positive only in the entorhinal cortex from those with tau extending into the lateral temporal (meta-temporal) regions. For amyloid PET, Centiloid values were used with the specified thresholds for each tracer; however, cases with Centiloid values between 10 and 18 for FBB and 10 and 20 for FBP were classified as borderline based on prior studies. Longitudinally, we assessed all instances of A and T reversion from positive to negative in CSF and PET separately in two groups, A+ to A− and T+ to T− to identify potential pathology reversion in cases with no recorded history of participation in amyloid- or tau-targeted clinical trials.

#### Discrepancy and discordance

We identified individuals with apparent discrepancies between their clinical classification and clinical rating scales (i.e. CDR-SB, MMSE and ADAS-cog 13). For example, (i) CN diagnosis with a CDR-SB score ≥ 2; (ii) CN diagnosis with a CDR-SB score > 1 and MMSE < 28; (iii) CN diagnosis with an ADAS-Cog 13 > 16 and CDR-SB = 0.5; and (iv) MCI diagnosis with a CDR-SB of zero and an MMSE score > 28 (Table 3).

**Table 3:** Discrepancies between clinical diagnosis and cognitive test outcomes

Variables	CN with CDR-SB > 1 and MMSE < 28	CN with CDR-SB ≥ 2	CN with ADAS-cog > 16 and CDR-SB = 0.5	MCI with CDR-SB = 0 and MMSE > 28
*N*	9	26	23	63
Baseline Age, years	79.4 ± 4.7	75.9 ± 5.6	75.5 ± 6	72 ± 7.7
Follow-up, years	7.8 ± 3.5	7.2 ± 3.9	6.8 ± 3.9	6.5 ± 3.7
Sex (F), *N*, %	1, 11.1%	11, 42.3%	9, 39.1%	29, 46%
Last ADAS-cog 13	21.6 [11.4–31.8]	18.2 [11.6–24.8]	18.6 [16.3–21]	12.6 [10.8–14.4]
Last MMSE	24.9 [21.5–28.3]	26.5 [24.3–28.7]	26.9 [25.9–27.8]	28.7 [28.3–29.1]
Last CDR	0.8 [0.4–1.3]	0.7 [0.4–0.9]	0.4 [0.2–0.5]	0.2 [0.1–0.3]
Last CDR-SB	4.7 [1.1–8.3]	3.5 [1.9–5.1]	1.3 [0.6–2]	0.7 [0.4–0.9]
A+, *N*, %	7, 87.5%	15, 71.4%	11, 55%	34, 57.6%
T+, *N*, %	3, 60%	9, 50%	12, 75%	36, 69.2%

Participants were classified into discordant groups based on inconsistencies between clinical diagnosis and cognitive test scores; no formal statistical comparisons were performed between groups.

Baseline age and follow-up duration = years (mean ± SD); Sex (F) = number of females (%); Last ADAS-Cog 13 = Alzheimer’s Disease Assessment Scale–Cognitive Subscale (13-item), mean (95% CI); Last MMSE = Mini-Mental State Examination score, mean (95% CI); Last CDR = Clinical Dementia Rating score, mean (95% CI); Last CDR-SB = CDR Sum of Boxes score, mean (95% CI); A+ = number of amyloid-positive participants (%); T+ = number of tau-positive participants (%).

We also evaluated the discordance between cross-sectional CSF and PET measures obtained within 1 year for a given individual: (i) discordant tau CSF and tau PET (CSF+/PET−, CSF−/PET+); and (ii) discordant amyloid CSF and amyloid PET (CSF+/PET−, CSF−/PET+), to identify heterogeneity and discrepancies between CSF and PET positivity (Table 4). To examine whether sex influenced concordance or discordance patterns, we performed an additional subject-level analysis in which each participant was classified based on the earliest discordant event observed across visits. A logistic regression model was used to evaluate the association between sex and overall discordance, and a multinomial regression model was used to assess associations with specific discordance phenotypes. All models were adjusted for baseline age and *APOE* ε4 carrier status.

**Table 4:** A/T biomarker variability

Variables	A/T positivity retrogression		Discordant Tau CSF and Tau PET		Discordant Amyloid CSF and Amyloid PET	
	A+ to A−	T+ to T−	CSF+/PET−	CSF−/PET+	CSF+/PET−	CSF−/PET+
*N* of cases	104	106	58	101	142	119
Baseline Age, years	70.9 ± 7.6	71.7 ± 6.8	72.8 ± 8	71.9 ± 5.9	70.6 ± 7.4	72.9 ± 6.9
Follow up, years	5.3 ± 3.3	5.2 ± 3.1	6 ± 4.1	4.7 ± 3.7	5.5 ± 3.8	5.7 ± 3.5
Female, *N*, %	41, 39.4%	50, 47.2%	25, 43.1%	50, 49.5%	57, 40.1%	76, 63.9%
APOE e4 carrier, *N*, %	28, 27.2%	37, 35.6%	18, 19.9%	27, 31%	33, 24.8%	42, 35.9%
Last ADAS-cog 13	13.4 [11.1–15.6]	15.2 [12.7–17.7]	11.9 [9.7–14.1]	18.5 [15.7–21.2]	14.5 [12.5–16.6]	14.3 [12.3–16.2]
Last MMSE	27.6 [26.9–28.3]	27 [26.2–27.9]	27.7 [27.1–28.4]	26.1 [25.1–27.1]	27.4 [26.7–28]	27.5 [26.9–28.2]
Last CDR	0.3 [0.2–0.4]	0.4 [0.3–0.5]	0.2 [0.2–0.3]	0.5 [0.4–0.7]	0.3 [0.3–0.4]	0.3 [0.2–0.4]
Last CDR-SB	1.5 [0.9–2]	2.1 [1.5–2.8]	1.1 [1–1.6]	2.7 [1.9–3.5]	1.7 [1.2–2.3]	1.7 [1.2–2.3]
CSF AB-42	935 [867–1002]	1276 [1117–1435]	1478 [1292–1664]	950 [852–1048]	773 [746–800]	1344 [1269–1420]
CSF p-tau181	18.15 [16.39–19.9]	27.01 [24.98–29.04]	32.10 [30.11–34.09]	18.09 [17.32–18.87]	16.34 [15.01–17.67]	31.08 [28.54–33.62]
Entorhinal Tau-PET SUVR	1.16 [1.09–1.24]	1.19 [1.17–1.22]	1.13 [1.1–1.15]	1.34 [1.31–1.38]	1.17 [1.12–1.22]	1.25 [1.15–1.34]
Meta-temporal Tau-PET SUVR	1.20 [1.12–1.28]	1.21 [1.19–1.23]	1.17 [1.14–1.21]	1.35 [1.31–1.39]	1.19 [1.14–1.23]	1.29 [1.20–1.38]
Amyloid, PET Centiloid	8.3 [4.6–12.1]	25.3 [16.9–33.7]	31.7 [22.2–42.2]	43.2 [35.1–51.3]	2.7 [1–4.4]	47 [41.8–52.2]

Participants were categorized into groups reflecting longitudinal biomarker reversion (A+→A− and T+→T−) and cross-modality discordance between CSF and PET biomarkers for tau and amyloid.

Baseline age and follow-up duration = years (mean ± SD); Sex (F) = number of females (%); APOE ε4 carrier = number of carriers (%); Last ADAS-Cog 13 = Alzheimer’s Disease Assessment Scale–Cognitive Subscale (13-item), mean (95% CI); Last MMSE = Mini-Mental State Examination score, mean (95% CI); Last CDR = Clinical Dementia Rating score, mean (95% CI); Last CDR-SB = CDR Sum of Boxes score, mean (95% CI); CSF Aβ42 = cerebrospinal fluid amyloid-β42, mean (95% CI); CSF p-tau181 = cerebrospinal fluid phosphorylated tau-181, mean (95% CI); Amyloid PET Centiloid = global cortical amyloid burden, mean (95% CI); Entorhinal Tau-PET SUVR = tau standardized uptake value ratio in the entorhinal cortex, mean (95% CI); Meta-Temporal Tau-PET SUVR = tau standardized uptake value ratio in the meta-temporal region, mean (95% CI).

### Statistical analysis

All analyses were performed using Python (version 3.11.5). Continuous variables were first assessed for normality using the Shapiro-Wilk test. Normally distributed continuous data were compared across diagnostic trajectory groups (stable, progressive, reversive, fluctuating) using ANOVA. For *post hoc* pairwise group comparisons, Tukey’s Honestly Significant Difference (HSD) test was applied, reporting mean differences, confidence intervals and adjusted *P*-values. Non-normally distributed continuous data were compared using the Kruskal-Wallis test followed by pairwise *post hoc* comparisons with correction for multiple testing. Categorical data were compared using Pearson’s chi-square test. If expected category sizes were small, Fisher’s exact test was used. *Post hoc* pairwise comparisons between categorical groups were performed with adjusted *P*-values. All statistical tests were two-tailed, with significance defined as α = 0.05 after correction for multiple comparisons. *P*-values are reported throughout, and 95% confidence intervals are provided for effect estimates. Summary statistics for outcomes, including mean, standard deviation and interquartile range, are reported in the Results and Supplementary Tables 1–3.

## Results

### Cohort characteristics

We included 2430 unique participants from four ADNI cohorts, as follows: cases with the original inclusion phase of ADNI 1 (*n* = 819); ADNI 2 (*n* = 790); ADNI GO (*n* = 131); ADNI 3 (*n* = 690). The mean age at baseline, when a participant initially enrolled in an ADNI cohort, ranged from 75.2 ± 6.8 years in ADNI 1 to 70.7 ± 7.4 years in ADNI 3. The duration of follow-up from initial enrolment varied across cohorts. The most recent ADNI 3 cohort had the shortest follow-up, averaging 2.2 ± 1.7 years. The original ADNI 1 cohort had the longest follow-up period, extending to 17 years, as their continuation across cohorts allowed for extended observation. Prevalence of *APOE* ε4 carriage ranged across phases (48.8% in ADNI 1 versus 40.6% in ADNI GO, Table 1).

### Distinct clinical trajectories and associated demographic, genetic, cognitive, psychiatric and treatment profiles

Four distinct trajectories were established based on longitudinal changes in cognitive impairment (CN, MCI and dementia): stable, progressive, reversive and fluctuating, among cases with a baseline and at least 2 years of follow-up visits (Table 2). Participants who reverted from MCI to CN (*n* = 57) were younger at baseline, with a mean age of 68.2 ± 6.8 years, compared to the group that progressed from MCI to dementia, with a mean age of 74 ± 7 years. Individuals with MCI at baseline who reverted to CN, compared to stable MCI and progressive MCI cases, had a lower prevalence of positive A and T cases at the time of their first available amyloid (42.3% versus 47.8% versus 88.2%) and tau measures (22.9% versus 42.6% versus 75.5%). In the same longitudinal MCI subgroups, ADAS-Cog 13 scores at baseline were significantly lower in the group that reverted compared to the stable group and the group that progressed: 9.2 (95% CI: 8.2, 10.2), 13.9 (95% CI: 13.3–14.4) and 20.4 (95% CI: 19.7–21). Stable CN had a lower prevalence of affective/behavioural symptomatology than both Stable MCI (25.9% versus 38.6%; OR = 0.56, 95% CI 0.39–0.78; *n* = 320 versus 295; adj. *P* < 0.001) and Stable Dementia (25.9% versus 43.7%; OR = 0.45, 95% CI 0.30–0.68; *n* = 320 versus 142; adj. *P* < 0.001).

Cases that fluctuated over time showed clinical assessment scores close to the thresholds between normal and early abnormal function, i.e. 12.4 (95% CI: 11–13.8), 28.2 (95% CI: 27.8–28.6) and 1.09 (95% CI: 0.8–1.3) for baseline ADAS-Cog 13, MMSE and CDR-SB, respectively. Among fluctuating cases, bidirectional transitions were observed; most fluctuating individuals developed clinical impairment over time. Specifically, 60 of 80 cases (75%) ended follow-up in MCI or dementia. Among those starting as MCI, only eight (14.8%) reverted to CN, whereas 13 (24.1%) progressed to dementia and 33 (61.1%) remained MCI. Of 1275 cases with available history, 469 participants (36.7%) had affective symptoms during or before data collection. A history of psychiatric symptoms was prevalent across all diagnostic trajectories, notably in MCI to dementia converters (43.5%), cases with fluctuations (42.7%) and dementia to MCI regressors (50%), and least common in the stable CN group (25.7%). Psychotropic medications, including antidepressants, were most frequently prescribed in the progressive category (42.6%) and cases with fluctuations (35%) (Table 2).

### Discrepancies between clinical syndrome and cognitive assessments

Among the filtered participants, 121 (5%) had discrepancies between syndromic diagnoses and cognitive test performance (Table 3). In the CN group, nine individuals exhibited CDR-SB > 1 and MMSE < 28, 26 had CDR-SB ≥ 2 and 23 showed ADAS-Cog 13 scores > 16. Conversely, 63 participants diagnosed with MCI maintained high functional and cognitive performance, with CDR-SB = 0 and MMSE >28. Detailed information about the demographics, clinical trajectories and amyloid and tau status of these groups is shown in Table 3. Baseline mean ages across groups ranged from 72 (95% CI: 70.1–73.9) to 79.4 (95% CI: 75.9–83) years, with follow-up durations between 6.5 and 7.8 years. Participants classified as CN at baseline but showing mild functional decline (CDR-SB > 1 with MMSE < 28 or CDR-SB ≥ 2) were more likely to experience further decline, with mean CDR-SB scores rising to 4.7 and 3.5 at the latest follow-up. Notably, 66% of these individuals retained the CN label despite this decline. By contrast, CN participants with baseline CDR-SB < 1.0 showed minimal cognitive change over time (mean CDR-SB of 0.03 in stable CN and 0.1 in those who progressed to MCI).

Importantly, some individuals categorized as CN at baseline showed impaired cognitive test scores despite their CN classification. These CNs with abnormal cognitive assessments (CDR and MMSE) exhibited higher rates of amyloid positivity (A+), ranging from 55–87.5% (Table 3), compared to 36.8% in stable CN individuals with normal cognitive scores (Table 2). A similar pattern was observed for tau positivity (T+), with rates between 50% and 75% in this group versus 31% in stable CN participants with normal cognitive function. Conversely, individuals initially classified as MCI but presenting with normal cognitive test scores (CDR-SB = 0, MMSE > 28) showed minimal decline during follow-up, with a mean CDR-SB of 0.7, a change that has debatable clinical meaningfulness.

### Descriptive analysis of CSF and PET

#### CSF pTau and tau PET positivity discordance

For 504 cases, we had both CSF and PET measures for tau obtained within a 1-year time interval. Figure 2 illustrates the % discordance between CSF p-tau181 and tau PET positivity status in the entorhinal and meta-temporal regions, employing two thresholding methods: the Youden threshold, which is more sensitive, and the mean SD threshold, which is more specific. Using the Youden threshold, tau PET identified 79 (15.7%) cases in the entorhinal region and 62 (12.3%) cases in the meta-temporal regions as T+, while CSF p-tau181 was classified as T−. In tau PET, using the mean SD threshold, there were fewer discordant PET and CSF cases. We identified 37 (7.3%) cases in the entorhinal region and 21 (4.2%) cases in the meta-temporal regions with a T+ classification, where CSF p-tau181 was classified as T−. These observations demonstrate that tau positivity, as assessed by CSF and PET, depends on the selected threshold, and there is no consistent precedence of CSF versus PET positivity. Sex was not associated with overall tau discordance (adjusted OR = 0.80, 95% CI 0.54–1.18, *P* = 0.27). When the two discordance categories were examined separately, no significant differences were observed for CSF+/PET− (OR = 0.71, *P* = 0.24) or CSF−/PET+ (OR = 0.86, *P* = 0.52) compared to concordant cases. These findings indicate that tau discordance patterns were not influenced by sex in this cohort.

**Figure 2:**
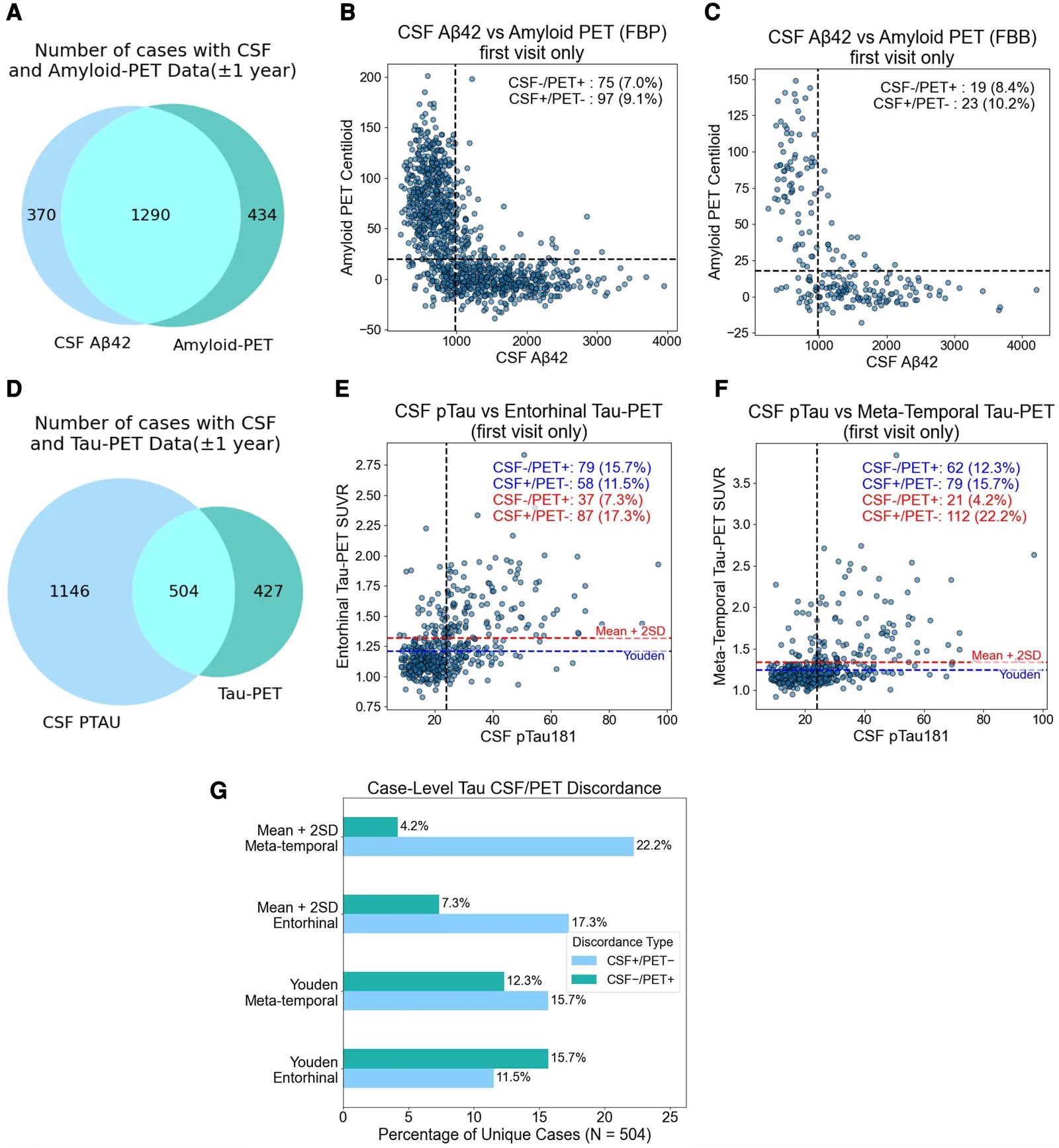
**Positivity concordance between baseline CSF and PET amyloid and tau biomarkers in participants with both measures available.** (**A**) Number of participants with available CSF amyloid beta 42 (Aβ42) or amyloid PET measures. (**B** and **C**) Concordance between CSF Aβ42 and amyloid Florbetapir (FBP, *N* = 1065) or Florbetaben (FBB, *N* = 225) PET Centiloid values, with dashed threshold lines indicating CSF and PET amyloid positivity and quadrants representing concordant and discordant biomarker classifications (CSF+/PET+, CSF−/PET−, CSF−/PET+ and CSF+/PET−). Each data point represents one unique case with both CSF and amyloid PET measures available. Using Spearman rank correlation, CSF Aβ42 was inversely associated with amyloid PET centiloid for both FBP (Spearman ρ = −0.68, *P* < 0.001) and FBB (Spearman ρ = −0.64, *P* < 0.001). (**D**) Number of participants with available CSF phosphorylated tau 181 (p-tau181), tau PET or both measures. (**E** and **F**) Concordance between CSF p-tau181 and tau PET standardized uptake value ratio (SUVR) in the entorhinal and meta-temporal regions among unique cases with both CSF and PET measures available (*N* = 504). Each data point represents one unique case. Dashed threshold lines indicate CSF p-tau181 positivity and tau PET positivity using two threshold definitions: the Youden index, with higher sensitivity for positivity, and mean plus two standard deviations (mean + 2SD), with higher specificity for a more defined tau pathology. CSF ptau181 was positively associated with tau PET SUVR in both the entorhinal region (Spearman -ρ = 0.49, *P* < 0.001) and meta-temporal region (Spearman ρ = 0.44, *P* < 0.001) with Spearman correlation. (**G**) Summary of tau biomarker positivity/negativity classification discordance across entorhinal and meta-temporal tau PET regions, showing the relative frequency of CSF+/PET− and CSF−/PET+ discordance under different tau PET regional definitions and positivity thresholds using the baseline time point for every unique case. The change in paired CSF/PET tau classification distribution (CSF−/PET−, CSF−/PET+, CSF+/PET− and CSF+/PET+) between the Youden and mean + 2SD tau PET thresholds was assessed using the Stuart-Maxwell test of marginal homogeneity. The distribution differed significantly between thresholds for both the entorhinal region (χ^2^ = 71.0, *P* < 0.001) and the meta-temporal region (χ^2^ = 74.0, *P* < 0.001). In specific category comparisons, all four CSF/PET categories changed significantly from Youden to mean + 2SD thresholds in both regions (all adjusted *P* < 0.001). Specifically, the mean + 2SD threshold increased CSF+/PET− discordance while decreasing CSF−/PET+ discordance in both entorhinal and meta-temporal tau PET regions.

When using the Youden threshold, 58 (11.5%) cases showed discordance between tau CSF and tau PET positivity, where tau CSF was positive, and tau PET exhibited negativity in both the entorhinal and Meta-temporal regions. Also, 101 (20%) cases showed CSF negativity, while PET was positive in entorhinal or Meta-temporal regions. Interestingly, the CSF p-tau181 in the CSF−/PET+ group was 18.09 (95% CI: 17.32–18.87), which was not close to the tau CSF threshold for positivity. At the time of discordance, both groups showed high amyloid PET values: 31.7 (95% CI: 22.2–42.2) in CSF+/PET−, and 43.2 (95% CI: 35.1–51.3) in the CSF−/PET+ group (Table 4).

### Discordance between entorhinal and meta-temporal positivity

Analysis of 931 cases with available Tau-Flortaucipir PET, using the Youden index as an inclusive threshold, revealed Tau positivity in the entorhinal region in 175 instances across 131 (14.1%) cases, while Tau was negative in the meta-temporal region. Additionally, we observed entorhinal region tau negativity alongside positive meta-temporal tau in 67 instances across 52 (5.6%) cases. Individuals who became entorhinal-positive before meta-temporal positivity [Ent+/Tem−, *n* = 131, ADAS-Cog13 mean = 13.5, 95% CI (12.0–15.0)] showed poorer cognitive performance at follow-up compared with those who were meta-temporal-positive first [Ent−/Tem+, *n* = 52, mean = 10.7, 95% CI (8.7–12.8); *P* = 0.035]. This effect was most pronounced for ADAS-Cog 13, whereas differences in MMSE were smaller (27.8 versus 28.5; *P* = 0.049), and no significant group differences were observed for CDR-SB or CDR.

CSF p-tau was significantly lower in individuals who were meta-temporal–positive but entorhinal negative at the same visit [Ent−/Tem+, *n* = 27, mean = 18.7 pg/mL, 95% CI (16.4–20.9)] compared with those who were entorhinal-positive but meta-temporal negative [Ent+/Tem−, *n* = 73, mean = 23.0 pg/mL, 95% CI (20.8–25.2); *P* = 0.009]. CSF Aβ42 was lower [1182 pg/mL (95% CI 1016–1348), *n* = 74 versus 1423 pg/mL (95% CI 1180–1666), *n* = 27; *P* = 0.11] and amyloid PET centiloids were higher [28.0 (95% CI 21.5–34.5), *n* = 128 versus 21.9 (95% CI 12.5–31.3), *n* = 51; *P* = 0.30] in the Ent+/Tem− group compared with Ent−/Tem+, but these differences did not reach statistical significance.

#### CSF amyloid and amyloid PET discordance

A total of 142 cases demonstrated discordant amyloid biomarker results, showing positive CSF Aβ42 levels but negative amyloid PET scans performed within 1 year of the CSF collection (Table 4). The mean Centiloid value for this group was notably low, at 2.7 (95% CI 1.0–4.4), indicating minimal amyloid deposition detectable by PET imaging. Clinically, these individuals exhibited mild cognitive decline over time, with a mean CDR-SB increasing from 0.3 at baseline to 1.7 at the most recent follow-up. Both CSF and PET tau levels remained low in this group (Table 4).

Conversely, in the CSF−/PET+ group (*n* = 119), tau levels were elevated on both CSF and PET measures, and a higher proportion were *APOE* ε4 carriers (35.9% versus 24.8% in the CSF+/PET− group). Despite this, the two groups were otherwise similar in terms of age and cognitive trajectory. Importantly, mean CSF Aβ42 levels in the CSF−/PET+ group with 95% CI ranged from 1269 to 1420 pg/mL, with a group mean of 1344 pg/mL, was well above the commonly accepted threshold for CSF amyloid positivity.

Sex was not associated with overall amyloid discordance (adjusted OR = 1.08, 95% CI 0.82–1.42, *P* = 0.60). However, when the two discordance categories were examined separately, divergent patterns emerged. Females were less likely to exhibit CSF+/PET− discordance (OR = 0.65, 95% CI 0.45–0.93, *P* = 0.020) and more likely to exhibit CSF−/PET+ discordance (OR = 1.99, 95% CI 1.33–2.98, *P* < 0.001) compared to concordant cases.

### Variability and discordance in A and T biomarker profiles

Among the 2094 participants with available data for amyloid classification, 104 (5%) individuals changed from A+ to A−. They were characterized by a lower percentage of *APOE* ε4 carriers and a lower mean centiloid PET value compared to the whole cohort (i.e. 27.2% versus 45.8%, and 8.3 Cl versus 36 Cl). Furthermore, they had low tau levels and milder decline on the CDR: 0.3 compared to the overall group (Table 4).

Among the entire ADNI data available for tau classification, there were 106 (5.1%) cases that changed from T+ to T−. At the time of this regression, tau levels were close to the threshold in CSF p-tau181, Entorhinal Tau-PET SUVR and Meta-Temporal tau-PET SUVR, which measured 27.01 (95% CI: 24.98–29.04), 1.19 (95% CI: 1.17–1.22), and 1.21 (95% CI: 1.19–1.23), respectively.

## Discussion

Alzheimer’s disease exhibits significant heterogeneity in clinical presentation, stemming from the existence of various disease subtypes and the presence of mixed pathologies among individuals. This diversity contributes to phenotypic variation and produces inconsistent disease trajectories, thereby leading to temporal heterogeneity throughout the course of the illness (Fig. 3).^17^ Meta-analyses suggest that approximately 18–30% of individuals diagnosed with MCI may return to normal cognitive function after a year or more,^18,19^ and these individuals demonstrate a decreased risk of progression to dementia compared to those with persistent MCI.^20-22^ Emerging research highlights distinct patterns of amyloid-β (Aβ) and tau deposition, cortical atrophy and dynamic changes in fluid biomarkers, all influenced by genetic, environmental and non-Alzheimer’s disease-related factors.^23,24^ The assumption that tau and amyloid marker positivity in CSF necessarily precedes changes seen in PET imaging is not consistently valid, as CSF biomarker levels can fluctuate during follow-up or even at different times of the day.^25-28^ While PET-CT imaging provides the most accurate representation of brain amyloid and tau pathology with minimal fluctuation, longitudinal studies have noted reductions in amyloid or tau load in subsequent scans of untreated individuals, potentially due to technical factors or progressive grey matter loss.^29,30^ Although plasma biomarkers offer advantages in cost-effectiveness and accessibility compared to CSF and imaging, their correlation with PET measures of amyloid and tau tends to be weaker. PET imaging remains more closely aligned with clinical diagnostic criteria and is integral to the management of Alzheimer’s disease and related neurodegenerative disorders.^31-33^

**Figure 3:**
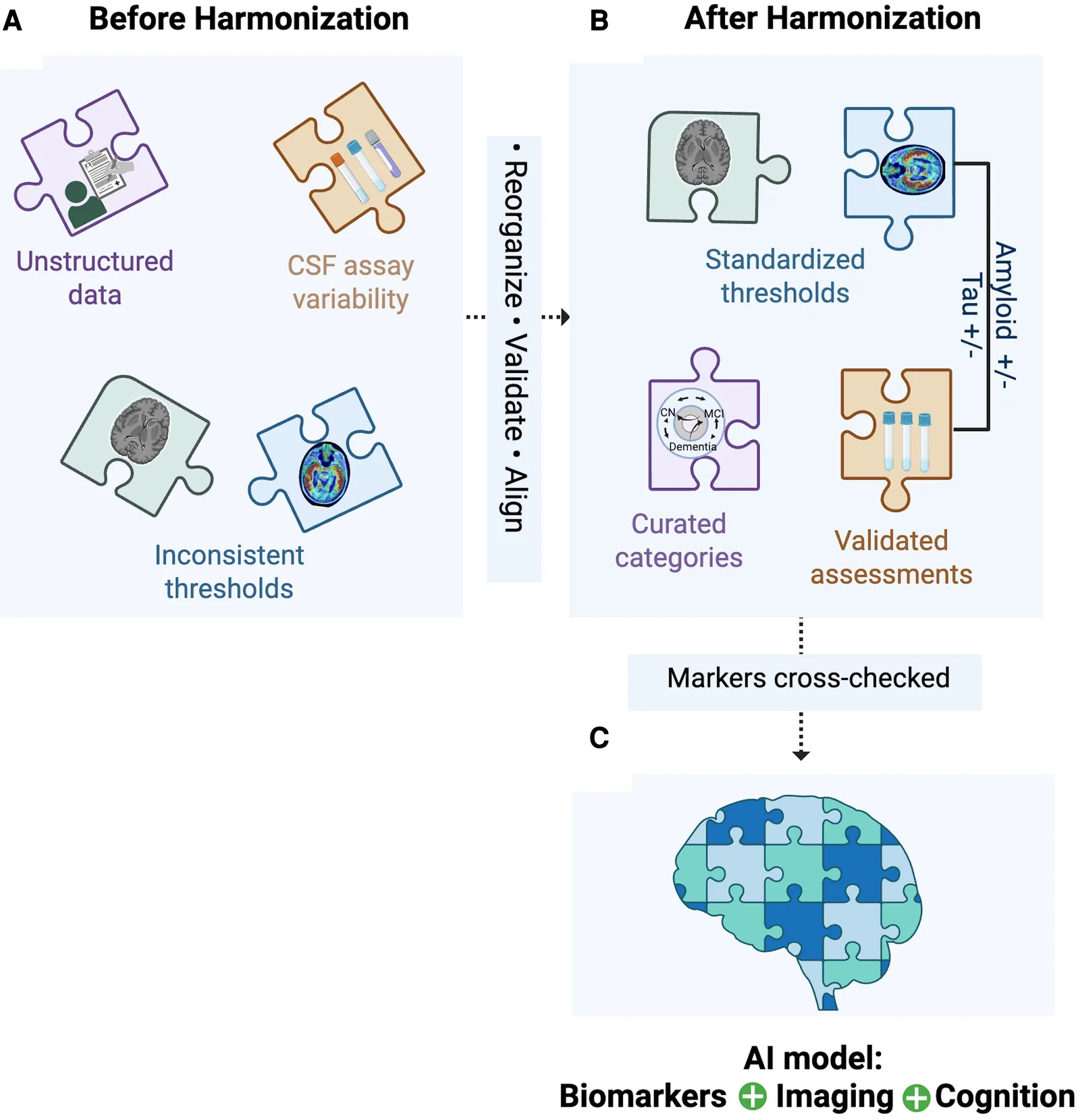
**Transforming fragmented Alzheimer's disease data into diagnostic frameworks for complex pathology and symptom mixtures.** (**A**) Before input standardization, diagnosis based on a single biomarker, considering assay and threshold variability, can lead to fragmented or discordant disease classification. This may dilute the actual Alzheimer's disease continuum classification. (**B**) A potential harmonization workflow includes reorganization, validation, and alignment of clinical variables, cognitive measures, CSF/PET biomarkers and stages and MRI-derived variables. The expected output of this multi-modal harmonization includes standardized thresholds, curated diagnostic categories beyond syndromic diagnosis and validated assessments of amyloid and tau positivity, while accounting for unique phenotypes arising from mixed pathologies. Cross-checking biomarker, imaging, cognitive and clinical features avoids tunnel vision on a single marker. (**C**) Downstream application of harmonized multi-modal data for building clinically feasible artificial intelligence models, with the goal of supporting individualized diagnosis and patient stratification. This framework may improve the reliability of models built using large cohorts already collected for Alzheimer's disease and related dementias. Created in BioRender. Fotouhi, M. (2026) https://BioRender.com/royy9hi

There is a clear and growing need to recognize the sources of variability and heterogeneity inherent in data collected from large cohorts in the field of Alzheimer’s disease. To fully leverage these datasets, it is essential to harmonize and standardize the definitions of the various stages of Alzheimer’s disease, whether through clinical diagnoses or biomarker assessments, and to better understand discrepancies among different diagnostic tests, biomarkers and the temporal fluctuations of these measures (Fig. 3).^34,35^ The implementation of clinical trials targeting different pathological aspects of the Alzheimer’s disease continuum, including amyloid and tau, further underscores the need for comprehensive and refined models to account for the diverse manifestations of the disease.^35,36^ Although awareness of heterogeneity in Alzheimer’s disease research is increasing, this study provides the most comprehensive analysis to date, using all ADNI cases with A/T classification, to examine cross-sectional and longitudinal discrepancies across clinical, cognitive and pathological biomarkers. We aimed to characterize these patterns while promoting data sharing and harmonization, thereby enabling the use of advanced machine learning approaches for more accurate diagnosis of Alzheimer’s disease, related dementias and their mimics.

Co-pathologies and non-degenerative phenocopies substantially contribute to the atypical and heterogeneous trajectories observed. We identified cognitive courses ranging from stability and progression to improvement, with some MCI cases reclassified as CN. Such regression often reflected initial overdiagnosis in borderline cases or treatable co-pathologies, particularly psychiatric symptoms. Notably, individuals who regressed from MCI to CN generally remained stable at follow-up, and cognitive gains were sometimes linked to treatment with antidepressants or behavioural interventions.^13^ Depression and other psychiatric symptoms frequently co-occur with early Alzheimer’s disease symptoms, complicating diagnostic accuracy.^13,14^ Diagnostic reversals may also occur due to practice effects in repeated testing^37^ or biases from recruitment constraints requiring fixed numbers across diagnostic categories, which can limit generalizability. Biomarker profiles supported these patterns: MCI to CN group had lower amyloid and tau positivity than stable MCI or progressive MCI, suggesting non-Alzheimer’s disease pathologies in their initial diagnoses. Participants with both improvement and decline formed a ‘fluctuating’ group, resembling stable MCI at baseline but showing less longitudinal decline, lower tau positivity (34.7% versus 45.9%) and higher prevalence of psychiatric symptoms. Such variability is also seen in other conditions, notably Lewy body dementia.^38^

In terms of the magnitude of biomarker discordance, CSF showed greater variability than PET, with some cases shifting from positive to negative or vice versa at follow-up. Such fluctuations, widely reported in prior studies,^15,35,36^ complicate treatment decisions when eligibility is based on CSF results, which may not fully reflect PET findings or true disease burden. Our findings align with prior evidence that agreement between CSF and PET is incomplete, particularly for tau. Bucci *et al*.^39^ reported moderate concordance for amyloid (κ ≈ 0.39–0.71) but generally fair concordance for tau (κ ≤ 0.40), with isolated CSF pTau positivity occurring more often than isolated tau PET positivity and isolated tau PET positivity being rare (∼0.7%).

Longitudinal reversion from positivity to negativity in CSF amyloid or tau do not necessarily indicate treatment efficacy, since marker negativity can occur independently of intervention. Laboratory methods and assay kits add further variability,^37^ although this study minimized such effects by using re-measured and batch-harmonized values. Nonetheless, factors such as specimen collection technique and time of day, unrecorded in these cohorts, are known to affect CSF amyloid and likely contributed to the variability that cannot be corrected retrospectively.^40^ These limitations highlight the need for standardized collection protocols, prospective validation and rigorous filtering when training AI models to ensure reliability. Amyloid PET values were harmonized to Centiloid scales, where 10–20 indicates borderline positivity, yet image quality, cortical atrophy and segmentation errors may still influence SUVR and longitudinal outcomes.^29,30^ For tau, PET and CSF discrepancies persist: CSF changes do not consistently precede PET changes, and tau PET patterns vary. PET did not uniformly demonstrate early entorhinal involvement before meta-temporal spread. Importantly, cases with early entorhinal tau showed faster cognitive decline and greater amyloid burden. These spatial differences underscore the heterogeneity within tau-positive cases, raising concerns about overgeneralizing positivity criteria and the specificity of flortaucipir for Alzheimer’s disease-related tau. Grouping all tau-positive participants together masks biologically distinct trajectories and undermines diagnostic precision, limiting the performance of AI-based disease models.

Although all initial diagnoses were based solely on baseline clinical assessments, the divergent trajectories observed over time suggest distinct underlying pathologies and co-pathologies. This underscores the limitations of relying solely on cross-sectional data or ‘snapshot’ diagnoses, which can misclassify heterogeneous cases under a single diagnostic label, thereby obscuring the true complexity of these disorders. To address these challenges, many prior studies have opted to exclude atypical cases, focusing instead on a narrow subset of patients who conform to expected disease progression pathways. While this strategy may support specific hypotheses, it fails to capture the full spectrum of real-world heterogeneity. Ironically, this exclusionary approach undermines the fundamental rationale for compiling large datasets: to leverage their diversity in building robust statistical and machine learning models for broader population-level insights. Our findings emphasize the importance of including and characterizing these atypical trajectories to better understand Alzheimer’s disease and related disorders in their full clinical complexity. The inclusion of indices of co-pathologies such as ischaemic changes,^41^ Lewy body^42^ and TDP-43 pathology^43^ in future studies, as these biomarkers are developed, will shed light on this heterogeneity. We emphasize that without an unbiased and systematic understanding of these conflicting results and atypical cases, harmonization efforts will remain incomplete and ineffective. Simply pooling large-scale datasets without addressing their inherent variability may obscure critical insights and hinder the development of robust, generalizable diagnostic and prognostic models. True integration across cohorts requires acknowledgement and resolution of these differences.

Several important limitations shape the interpretation and broader applicability of this study. The dynamic nature of cognitive decline in ageing means diagnostic categories, such as stable, progressive or reversive cognitive status, are inherently fluid and are further complicated by variable follow-up durations and pandemic-related disruptions, causing missed or delayed visits. These factors introduce challenges in assigning definitive trajectories, potentially influencing the proportion of cases in each diagnostic group. Additionally, retrospective elements of the study precluded standardized timing for sample collection and full characterization of comorbidities, medication usage and environmental confounders. Most notably, the cohort exhibits significant sampling bias: 94.8% of ADNI participants were non-Latino Caucasians, thereby underscoring a substantial gap in representation. This lack of diversity restricts the generalizability of findings and constrains our understanding of how genetic, ethnic and social backgrounds influence biomarker patterns, disease trajectories and risk profiles, especially as emerging research underscores differences in biomarker expression and disease course among underrepresented populations.^44,45^

To address these gaps, future research should prioritize inclusive recruitment to ensure representation across ethno-racial and socioeconomic groups, thereby improving the applicability and equity of analytical models and clinical guidelines. Expanding multi-modal data acquisition, including advanced MRI, plasma biomarkers, digital assessments and more granular psychiatric and comorbidity profiling, will enhance our ability to characterize and interpret disease heterogeneity. Standardizing protocols for data acquisition, analysis and reporting will be necessary across studies and phases to facilitate meaningful cross-cohort comparisons. Longitudinal and trajectory frameworks, rather than static, cross-sectional labels, are essential for capturing the evolving realities of cognitive decline and biomarker fluctuation. Finally, collaborative open science efforts that emphasize rigorous annotation, cross-cohort harmonization and transparent sharing of raw and processed data will be vital for the development of robust and generalizable AI diagnostic and prognostic models capable of supporting precision medicine in Alzheimer’s disease and related disorders.

As a next step, we aim to harmonize and recategorize cases with clinical and biomarker discrepancies, incorporating multi-modal information and release the harmonized version of the data to researchers in the field of Alzheimer’s disease globally. We will also extend our investigation to explore discrepancies within the MRI data collected in ADNI. Our long-term goal is to propose standardized approaches that address these inconsistencies, thereby enabling the creation of harmonized datasets that can be reliably used by researchers worldwide. Future phases of this work will also incorporate ADNI datasets from international cohorts, further contributing to the global effort towards standardized and interoperable Alzheimer’s research data.

## Conclusions

Reversions in syndromal diagnoses, fluctuating cognitive trajectories and biomarker discordance are present in the ADNI cohort and potentially are rooted in co-pathologies and psychiatric comorbidities. The use of standard measurement protocols did not eliminate fluctuations in CSF and PET biomarkers or resolve inconsistencies between syndromal classifications and cognitive test scores. These results highlight the inadequacy of relying solely on clinical rating scores or single-modality biomarker thresholds for disease definition and underscore the necessity of longitudinal, multi-modal and trajectory approaches. This work provides a foundation for advancing more precise diagnostic criteria, clinical trial recruitment and a deeper biological understanding of Alzheimer’s disease and related dementias.

## Supplementary Material

fcag312_Supplementary_Data

## Data Availability

The data utilized in this study were obtained from the Alzheimer's Disease Neuroimaging Initiative (ADNI). All primary data, including clinical records, cognitive performance metrics, CSF biomarkers, PET imaging data and relevant demographic and medical history information, are available to qualified researchers upon request through the ADNI data repository (http://adni.loni.usc.edu/). In addition, the harmonized dataset generated after this study will be made available to ADNI researchers through the platform to facilitate future research. The code used for analyses in this study is available on GitHub (https://github.com/MacMasterCor/ClassDiscordance.git).
