# Supplementary material for: Diagnostic classifications based on cognitive, CSF, and PET biomarkers across ADNI cohorts: cross-sectional and longitudinal variability
Source: Brain Commun. 2026 Aug 26;8(5):fcag312. doi: 10.1093/braincomms/fcag312 (PMC13648873; doi:10.1093/braincomms/fcag312)
Supplement: Supplementary file 1 [file fcag312_supplementary_data.pdf]

Supplementary Material

1. Supplementary Methods

Neurocognitive Evaluations and Clinical Diagnosis in ADNI

Cognitively normal participants are defined as having slight or no significant memory complaints beyond what is expected for their age, non-depressed, non-MCI, and non-demented, normal memory function adjusted for education level on the Logical Memory II subscale, having an MMSE score between 24 and 30, having a CDR of 0, no significant impairment in cognitive function or daily activities, and stability of permitted medications for at least 4 weeks prior to screening. Excluded medications include psychoactive drugs with anticholinergic side effects, while antidepressants and estrogen replacement therapy are allowed. MCI participants are characterized by subjective memory complaints corroborated by a study partner or clinician, non-depressed, and non-demented, abnormal memory function adjusted for education level, an MMSE score between 24 and 30, a CDR of 0.5, preserved general cognition and functional performance, no diagnosis of dementia or Alzheimer's disease, and similar medication guidelines as CN participants. Cholinesterase inhibitors and memantine are allowable if stable for 4 weeks prior to screening. AD dementia participants must exhibit subjective memory complaints corroborated by a study partner or clinician, abnormal memory function adjusted for education level, an MMSE score between 20 and 26, a CDR of 0.5 or above, meet criteria for probable AD as defined by the National Institute of Neurological and Communicative Disorders and Stroke-Alzheimer's Disease and Related Disorders Association (NINCDS-ADRDA), and follow similar medication guidelines as MCI participants.

CSF Biomarkers

CSF Ab42 and phosphorylated-Tau 181 (p-Tau) were analyzed by the electrochemiluminescence immunoassays Elecsys β-Amyloid(1-42) CSF and phospho-Tau(181P) CSF on a fully automated Elecsys cobas e 601 instrument and a single lot of reagents for each of the measured biomarkers [1–3]. The Roche Elecsys β-Amyloid (1-42) CSF and Elecsys Phospho-Tau(181P) CSF immunoassays were used following a Roche Study Protocol at the UPenn/ADNI Biomarker Laboratory, according to the kit manufacturer's instructions and as described in previous studies. In this study, we have implemented the latest homogenized batch analysis for all cases. The analyte measuring ranges were, from UPENN CSF Biomarkers Elecsys (BATCH 9-13), lower technical limit to upper technical limit for each biomarker: 200 to 1700 pg/mL for Elecsys β-Amyloid (1-42) CSF immunoassay, and 8 to 120 pg/mL for Elecsys Phospho-Tau (181P) CSF immunoassay.

PET Scan

Amyloid positivity was determined using PET data based on ADNI guidelines, which set a specific cutoff for amyloid-PET positivity at 18 Centiloid (CL) for ^{18}F-florbetaben (FBB) (1.08 SUVR) or 20 CL for ^{18}F-florbetapir (FBP) (1.11 SUVR), where a higher centiloid means more amyloid plaque burden[4]. The centiloid was quantified across cortical summary SUVRs from frontal, temporal, parietal, and cingulate regions, normalized to the whole cerebellum. Given the analysis

of two amyloid PET tracers, SUVR levels were converted to CL using the specific equation for each tracer provided by ADNI. Since only one tracer for tau PET was used in ADNI, [18F] flortaucipir (FTP), the SUVR values were used, and the positivity threshold established in the previous study was 1.21 in the entorhinal cortex and 1.25 in the meta-temporal ROI (entorhinal, amygdala, fusiform, inferior temporal, middle temporal), with higher SUVRs showing greater tau burden[5]. In order to reduce the influence of off-target binding in the dorsal cerebellum, regional SUVRs have been intensity normalized by the inferior cerebellar grey matter reference region[6]. Details of PET acquisition are described in previous publications[4–7] and on the ADNI website (https://adni.loni.usc.edu/).

Modality Availability and Missingness

To evaluate the potential impact of incomplete biomarker availability on A/T classification, we examined subject modality availability for amyloid and tau measures across the full cohort (N = 2430).

- Amyloid

Among 2430 participants, 1290 (53.1%) had both CSF Aβ42 and amyloid PET measures available within the predefined 1-year pairing window. An additional 326 (13.4%) had CSF measures only, and 390 (16.0%) had PET measures only. Forty-four participants (1.8%) had both modalities available but not within the 1-year pairing window, and 380 (15.6%) had neither modality available.

Discordance analyses reported in the main manuscript were restricted to the 1290 participants with paired CSF and PET measures within the 1-year window.

- Tau

For tau biomarkers, 503 of 2430 participants (20.7%) had both CSF pTau-181 and tau PET measures available within the 1-year window. A larger proportion had CSF measures only (985 participants, 40.5%), while 268 (11.0%) had PET measures only. An additional 162 participants (6.7%) had both modalities available but outside the pairing window, and 512 (21.1%) had neither modality available.

As with amyloid, tau discordance analyses were restricted to the 503 participants with paired CSF and PET measures within the 1-year window.

2. Supplementary Post hoc Analysis Results

Supplementary Table 1.

1.1 Baseline age

Tukey HSD Post Hoc Test Results for AGE by ADNI phase:
Multiple Comparison of Means - Tukey HSD, FWER=0.05

group1	group2	meandiff	p-adj	lower	upper	reject
ADNI1	ADNI2	-2.5098	0.0	-3.4274	-1.5922	True
ADNI1	ADNI3	-4.4901	0.0	-5.441	-3.5393	True
ADNI1	ADNIGO	-3.6495	0.0	-5.3809	-1.918	True
ADNI2	ADNI3	-1.9804	0.0	-2.9391	-1.0216	True
ADNI2	ADNIGO	-1.1397	0.3303	-2.8755	0.5962	False
ADNI3	ADNIGO	0.8407	0.6063	-0.913	2.5943	False

1.2.Follow-up years

Tukey HSD Post Hoc Test Results for FOLLOWUP_YEARS by ADNI phase:
Multiple Comparison of Means - Tukey HSD, FWER=0.05

group1	group2	meandiff	p-adj	lower	upper	reject
ADNI1	ADNI2	-0.1323	0.8601	-0.5644	0.2997	False
ADNI1	ADNI3	-2.6137	0.0	-3.0613	-2.166	True
ADNI1	ADNIGO	1.0878	0.0034	0.2727	1.903	True
ADNI2	ADNI3	-2.4813	0.0	-2.9327	-2.0299	True
ADNI2	ADNIGO	1.2202	0.0007	0.4029	2.0374	True
ADNI3	ADNIGO	3.7015	0.0	2.8759	4.5271	True

1.3.Sex

Pairwise Post Hoc Chi-Squared Tests (Bonferroni corrected) for sex:
ADNI1 vs ADNIGO: p = 0.4387, adjusted p = 1.0000
ADNI1 vs ADNI2: p = 0.0140, adjusted p = 0.0842
ADNI1 vs ADNI3: p = 0.0000, adjusted p = 0.0000
ADNIGO vs ADNI2: p = 0.7138, adjusted p = 1.0000
ADNIGO vs ADNI3: p = 0.0833, adjusted p = 0.4997
ADNI2 vs ADNI3: p = 0.0143, adjusted p = 0.0857

1.4.Cognitive test scores including ADAS-cog13, MMSE, CDR, CDR-SB

Tukey HSD Post Hoc Test Results by Variable:

- Variable: ADAS13

Multiple Comparison of Means - Tukey HSD, FWER=0.05

group1	group2	meandiff	p-adj	lower	upper	reject
ADNI1	ADNI2	-2.3538	0.0	-3.5476	-1.1601	True
ADNI1	ADNI3	-5.3122	0.0	-6.5506	-4.0738	True
ADNI1	ADNIGO	-6.0266	0.0	-8.2776	-3.7756	True
ADNI2	ADNI3	-2.9584	0.0	-4.2069	-1.7099	True
ADNI2	ADNIGO	-3.6728	0.0002	-5.9294	-1.4162	True
ADNI3	ADNIGO	-0.7144	0.8519	-2.9949	1.5661	False

```
- Variable: MMSE
Multiple Comparison of Means - Tukey HSD, FWER=0.05
===================================================
group1 group2 meandiff p-adj   lower  upper  reject
---------------------------------------------------
 ADNI1  ADNI2   0.6881    0.0  0.3543 1.0218   True
 ADNI1  ADNI3   1.1635    0.0  0.8175 1.5095   True
 ADNI1 ADNIGO   1.5465    0.0  0.9167 2.1763   True
 ADNI2  ADNI3   0.4755 0.0026  0.1266 0.8243   True
 ADNI2 ADNIGO   0.8584 0.0027  0.2271 1.4898   True
 ADNI3 ADNIGO    0.383 0.4117 -0.2549 1.0209  False
---------------------------------------------------

- Variable: CDR GLOBAL
Multiple Comparison of Means - Tukey HSD, FWER=0.05
====================================================
group1 group2 meandiff p-adj   lower   upper  reject
----------------------------------------------------
 ADNI1  ADNI2  -0.0474 0.0107 -0.0867  -0.008   True
 ADNI1  ADNI3  -0.1598    0.0 -0.2006 -0.1191   True
 ADNI1 ADNIGO   0.0836 0.0198  0.0094  0.1578   True
 ADNI2  ADNI3  -0.1125    0.0 -0.1536 -0.0714   True
 ADNI2 ADNIGO    0.131    0.0  0.0566  0.2054   True
 ADNI3 ADNIGO   0.2435    0.0  0.1683  0.3186   True
----------------------------------------------------

- Variable: CDRSB
Multiple Comparison of Means - Tukey HSD, FWER=0.05
====================================================
group1 group2 meandiff p-adj   lower   upper  reject
----------------------------------------------------
 ADNI1  ADNI2  -0.2518 0.0201 -0.4755  -0.028   True
 ADNI1  ADNI3  -0.7793    0.0 -1.0112 -0.5475   True
 ADNI1 ADNIGO  -0.5601 0.0037 -0.9822 -0.1379   True
 ADNI2  ADNI3  -0.5275    0.0 -0.7613 -0.2938   True
 ADNI2 ADNIGO  -0.3083 0.2402 -0.7315   0.115  False
 ADNI3 ADNIGO   0.2192 0.5514 -0.2083  0.6468  False
```

1.5.CSF Amyloid beta 42

```
Tukey HSD Post Hoc Test Results for CSF Amyloid beta 42 by ADNI phase:
 Multiple Comparison of Means - Tukey HSD, FWER=0.05
======================================================
group1 group2 meandiff p-adj   lower    upper   reject
------------------------------------------------------
 ADNI1  ADNI2 196.4198    0.0  99.0333 293.8064   True
 ADNI1  ADNI3 278.1156    0.0 167.3586 388.8726   True
 ADNI1 ADNIGO 358.9772    0.0 193.2301 524.7243   True
 ADNI2  ADNI3  81.6958 0.1512 -18.0126 181.4042  False
 ADNI2 ADNIGO 162.5574 0.0421   3.9802 321.1347   True
 ADNI3 ADNIGO  80.8616 0.5987 -86.2602 247.9835  False
```

1.6.CSF pTau

```
Tukey HSD Post Hoc Test Results for CSF pTau by ADNI phase:
Multiple Comparison of Means - Tukey HSD, FWER=0.05
====================================================
group1 group2 meandiff p-adj   lower   upper  reject
----------------------------------------------------
 ADNI1  ADNI2  -2.6443 0.0108 -4.8429 -0.4457   True
```

ADNI1	ADNI3	-5.8444	0.0	-8.3486	-3.3402	True
ADNI1	ADNIGO	-5.0365	0.003	-8.7684	-1.3045	True
ADNI2	ADNI3	-3.2001	0.0015	-5.4501	-0.9501	True
ADNI2	ADNIGO	-2.3922	0.311	-5.9586	1.1742	False
ADNI3	ADNIGO	0.8079	0.946	-2.9546	4.5704	False

1.7.Amyloid PET Centiloid

Tukey HSD Post Hoc Test Results for Amyloid PET CENTILOIDS by ADNI phase:
Multiple Comparison of Means - Tukey HSD, FWER=0.05

group1	group2	meandiff	p-adj	lower	upper	reject
ADNI1	ADNI2	6.2915	0.308	-3.0572	15.6402	False
ADNI1	ADNI3	-3.7738	0.7417	-13.3513	5.8036	False
ADNI1	ADNIGO	-3.4457	0.9107	-16.7989	9.9074	False
ADNI2	ADNI3	-10.0654	0.0003	-16.4668	-3.6639	True
ADNI2	ADNIGO	-9.7372	0.119	-21.0314	1.5569	False
ADNI3	ADNIGO	0.3281	0.9999	-11.1561	11.8123	False

1.8.Entorhinal tau PET SUVR

Tukey HSD Post Hoc Test Results for Entorhinal tau PET SUVR by ADNI phase:
Multiple Comparison of Means - Tukey HSD, FWER=0.05

group1	group2	meandiff	p-adj	lower	upper	reject
ADNI1	ADNI2	-0.0227	0.9426	-0.126	0.0807	False
ADNI1	ADNI3	-0.0221	0.937	-0.1193	0.0752	False
ADNI1	ADNIGO	-0.0319	0.9434	-0.178	0.1142	False
ADNI2	ADNI3	0.0006	1.0	-0.0521	0.0533	False
ADNI2	ADNIGO	-0.0092	0.9974	-0.1303	0.1119	False
ADNI3	ADNIGO	-0.0098	0.9963	-0.1258	0.1061	False

1.9.Meta-temporal tau PET SUVR

Tukey HSD Post Hoc Test Results for Meta-temporal tau PET SUVR by ADNI phase:
Multiple Comparison of Means - Tukey HSD, FWER=0.05

group1	group2	meandiff	p-adj	lower	upper	reject
ADNI1	ADNI2	0.0096	0.9963	-0.103	0.1221	False
ADNI1	ADNI3	0.0086	0.9968	-0.0972	0.1145	False
ADNI1	ADNIGO	-0.0036	0.9999	-0.1626	0.1555	False
ADNI2	ADNI3	-0.001	1.0	-0.0583	0.0564	False
ADNI2	ADNIGO	-0.0131	0.9941	-0.145	0.1187	False
ADNI3	ADNIGO	-0.0122	0.9946	-0.1384	0.114	False

Supplementary Table 2.

2.1.Baseline age

===== ONE-WAY ANOVA =====
F-statistic: 9.8940
P-value: 0.000000

===== Tukey HSD Post Hoc (FWER controlled) =====
Multiple Comparison of Means - Tukey HSD, FWER=0.05

group1	group2	meandiff	p-adj	lower	upper	reject
CN	CN→Dementia	2.766	0.3829	-1.0768	6.6088	False
CN	CN→MCI	2.9549	0.0098	0.4077	5.5021	True
CN	Dementia	3.1997	0.0	1.2934	5.1061	True
CN	Dementia→MCI	4.9902	0.9446	-7.3918	17.3722	False
CN	Fluctuation	-0.786	0.9901	-3.3604	1.7884	False
CN	MCI	0.3167	0.9992	-1.1766	1.8101	False
CN	MCI→CN	-4.2045	0.0005	-7.1934	-1.2156	True
CN	MCI→Dementia	1.5867	0.0367	0.0507	3.1228	True
CN→Dementia	CN→MCI	0.1889	1.0	-4.2191	4.5969	False
CN→Dementia	Dementia	0.4338	1.0	-3.6376	4.5051	False
CN→Dementia	Dementia→MCI	2.2242	0.9998	-10.6698	15.1182	False
CN→Dementia	Fluctuation	-3.552	0.2355	-7.9758	0.8717	False
CN→Dementia	MCI	-2.4492	0.5766	-6.3444	1.4459	False
CN→Dementia	MCI→CN	-6.9705	0.0001	-11.6476	-2.2933	True
CN→Dementia	MCI→Dementia	-1.1792	0.9909	-5.0909	2.7325	False
CN→MCI	Dementia	0.2449	1.0	-2.6356	3.1254	False
CN→MCI	Dementia→MCI	2.0354	0.9999	-10.5335	14.6042	False
CN→MCI	Fluctuation	-3.7409	0.0163	-7.101	-0.3807	True
CN→MCI	MCI	-2.6381	0.0478	-5.2636	-0.0126	True
CN→MCI	MCI→CN	-7.1594	0.0	-10.8467	-3.472	True
CN→MCI	MCI→Dementia	-1.3681	0.8031	-4.0181	1.2819	False
Dementia	Dementia→MCI	1.7905	1.0	-10.6643	14.2453	False
Dementia	Fluctuation	-3.9858	0.0007	-6.8903	-1.0812	True
Dementia	MCI	-2.883	0.0003	-4.8927	-0.8733	True
Dementia	MCI→CN	-7.4043	0.0	-10.6818	-4.1267	True
Dementia	MCI→Dementia	-1.613	0.2557	-3.6546	0.4286	False
Dementia→MCI	Fluctuation	-5.7762	0.8876	-18.3507	6.7982	False
Dementia→MCI	MCI	-4.6735	0.9625	-17.0718	7.7248	False
Dementia→MCI	MCI→CN	-9.1947	0.3706	-21.8605	3.471	False
Dementia→MCI	MCI→Dementia	-3.4035	0.9952	-15.807	9.0	False
Fluctuation	MCI	1.1028	0.9339	-1.5491	3.7547	False
Fluctuation	MCI→CN	-3.4185	0.0981	-7.1247	0.2877	False
Fluctuation	MCI→Dementia	2.3728	0.1302	-0.3034	5.0489	False
MCI	MCI→CN	-4.5212	0.0002	-7.5772	-1.4653	True
MCI	MCI→Dementia	1.27	0.2999	-0.3927	2.9327	False
MCI→CN	MCI→Dementia	5.7913	0.0	2.7143	8.8683	True

2.2.Follow-up years

```
===== ONE-WAY ANOVA =====
F-statistic: 34.2791
P-value: 3.402750e-50

===== Tukey HSD (FWER controlled) =====
Multiple Comparison of Means - Tukey HSD, FWER=0.05
```

group1	group2	meandiff	p-adj	lower	upper	reject
CN	CN→Dementia	3.7504	0.0	2.0815	5.4193	True
CN	CN→MCI	1.1205	0.0444	0.0142	2.2267	True
CN	Dementia	-2.6184	0.0	-3.4463	-1.7905	True
CN	Dementia→MCI	-2.5	0.8805	-7.8773	2.8774	False
CN	Fluctuation	2.5554	0.0	1.4373	3.6734	True
CN	MCI	-0.5437	0.1856	-1.1923	0.1048	False
CN	MCI→CN	1.3406	0.0368	0.0425	2.6386	True
CN	MCI→Dementia	-0.0361	1.0	-0.7032	0.631	False
CN→Dementia	CN→MCI	-2.6299	0.0007	-4.5443	-0.7156	True
CN→Dementia	Dementia	-6.3688	0.0	-8.1369	-4.6006	True
CN→Dementia	Dementia→MCI	-6.2503	0.0158	-11.8501	-0.6506	True
CN→Dementia	Fluctuation	-1.195	0.5915	-3.1162	0.7262	False
CN→Dementia	MCI	-4.2941	0.0	-5.9857	-2.6025	True
CN→Dementia	MCI→CN	-2.4098	0.0073	-4.4411	-0.3786	True
CN→Dementia	MCI→Dementia	-3.7864	0.0	-5.4853	-2.0876	True
CN→MCI	Dementia	-3.7389	0.0	-4.9898	-2.4879	True
CN→MCI	Dementia→MCI	-3.6204	0.5013	-9.079	1.8381	False
CN→MCI	Fluctuation	1.4349	0.0582	-0.0244	2.8942	False
CN→MCI	MCI	-1.6642	0.0002	-2.8044	-0.5239	True
CN→MCI	MCI→CN	0.2201	1.0	-1.3813	1.8215	False
CN→MCI	MCI→Dementia	-1.1565	0.0478	-2.3074	-0.0057	True
Dementia	Dementia→MCI	0.1184	1.0	-5.2906	5.5274	False
Dementia	Fluctuation	5.1738	0.0	3.9124	6.4352	True
Dementia	MCI	2.0747	0.0	1.2019	2.9475	True
Dementia	MCI→CN	3.959	0.0	2.5355	5.3824	True
Dementia	MCI→Dementia	2.5823	0.0	1.6957	3.469	True
Dementia→MCI	Fluctuation	5.0554	0.0955	-0.4056	10.5163	False
Dementia→MCI	MCI	1.9563	0.97	-3.4282	7.3407	False
Dementia→MCI	MCI→CN	3.8405	0.4269	-1.6601	9.3411	False
Dementia→MCI	MCI→Dementia	2.4639	0.89	-2.9228	7.8506	False
Fluctuation	MCI	-3.0991	0.0	-4.2508	-1.9474	True
Fluctuation	MCI→CN	-1.2148	0.3161	-2.8244	0.3948	False
Fluctuation	MCI→Dementia	-2.5915	0.0	-3.7537	-1.4292	True
MCI	MCI→CN	1.8843	0.0004	0.5571	3.2114	True
MCI	MCI→Dementia	0.5076	0.4168	-0.2144	1.2297	False
MCI→CN	MCI→Dementia	-1.3766	0.0377	-2.7129	-0.0403	True

2.3.APOEe4

Chi-squared test p-value: 2.3568e-25

Post-hoc pairwise Fisher exact tests (Holm correction):

Group1	Group2	OddsRatio	p_raw	p_adj_holm	significant_holm_0.05	Carrier_prop_direction
Stable_CN	MCI_to_Dementia	0.232853	2.080009e-22	7.488033e-21	True	Stable_CN (0.297) vs MCI_to_Dementia (0.644)
Stable_CN	Stable_Dementia	0.222539	5.892494e-16	2.062373e-14	True	Stable_CN (0.297) vs Stable_Dementia (0.655)
Stable_MCI	MCI_to_Dementia	0.330311	4.694655e-12	1.596183e-10	True	Stable_MCI (0.374) vs MCI_to_Dementia (0.644)
Stable_MCI	Stable_Dementia	0.315680	3.144247e-09	1.037601e-07	True	Stable_MCI (0.374) vs Stable_Dementia (0.655)
CN_to_MCI	MCI_to_Dementia	0.307979	4.275018e-06	1.368006e-04	True	CN_to_MCI (0.358) vs MCI_to_Dementia (0.644)
Stable_Dementia	CN_to_MCI	3.397459	1.920513e-05	5.953590e-04	True	Stable_Dementia (0.655) vs CN_to_MCI (0.358)
MCI_to_Dementia	Fluctuating	2.659628	1.725157e-04	5.175471e-03	True	MCI_to_Dementia (0.644) vs Fluctuating (0.405)
MCI_to_Dementia	MCI_to_CN	3.018018	1.944022e-04	5.637664e-03	True	MCI_to_Dementia (0.644) vs MCI_to_CN (0.375)
Stable_Dementia	Fluctuating	2.782895	3.035422e-04	8.499139e-03	True	Stable_Dementia (0.655) vs Fluctuating (0.405)
Stable_Dementia	MCI_to_CN	3.157895	3.035407e-04	8.499139e-03	True	Stable_Dementia (0.655) vs MCI_to_CN (0.375)
Stable_Dementia	CN_to_Dementia	2.571429	1.804762e-02	4.692381e-01	False	Stable_Dementia (0.655) vs CN_to_Dementia (0.424)
Stable_CN	Stable_MCI	0.704950	2.049106e-02	5.122766e-01	False	Stable_CN (0.297) vs Stable_MCI (0.374)
CN_to_Dementia	MCI_to_Dementia	0.406913	2.203885e-02	5.289324e-01	False	CN_to_Dementia (0.424) vs MCI_to_Dementia (0.644)
CN_to_MCI	Fluctuating	0.819111	6.257326e-01	1.000000e+00	False	CN_to_MCI (0.358) vs Fluctuating (0.405)
CN_to_MCI	Dementia_to_MCI	1.115385	1.000000e+00	1.000000e+00	False	CN_to_MCI (0.358) vs Dementia_to_MCI (0.333)
CN_to_Dementia	Fluctuating	1.082237	1.000000e+00	1.000000e+00	False	CN_to_Dementia (0.424) vs Fluctuating (0.405)
MCI_to_Dementia	Dementia_to_MCI	3.621622	2.931103e-01	1.000000e+00	False	MCI_to_Dementia (0.644) vs Dementia_to_MCI (0.333)
CN_to_MCI	MCI_to_CN	0.929487	8.584454e-01	1.000000e+00	False	CN_to_MCI (0.358) vs MCI_to_CN (0.375)
MCI_to_CN	Dementia_to_MCI	1.200000	1.000000e+00	1.000000e+00	False	MCI_to_CN (0.375) vs Dementia_to_MCI (0.333)
CN_to_Dementia	Dementia_to_MCI	1.473684	1.000000e+00	1.000000e+00	False	CN_to_Dementia (0.424) vs Dementia_to_MCI (0.333)
CN_to_Dementia	MCI_to_CN	1.228070	6.601633e-01	1.000000e+00	False	CN_to_Dementia (0.424) vs MCI_to_CN (0.375)
Stable_Dementia	MCI_to_Dementia	1.046347	8.409274e-01	1.000000e+00	False	Stable_Dementia (0.655) vs MCI_to_Dementia (0.644)
Stable_Dementia	Dementia_to_MCI	3.789474	2.821483e-01	1.000000e+00	False	Stable_Dementia (0.655) vs Dementia_to_MCI (0.333)
MCI_to_CN	Fluctuating	0.881250	8.581704e-01	1.000000e+00	False	MCI_to_CN (0.375) vs Fluctuating (0.405)
Stable_MCI	Fluctuating	0.878505	6.096212e-01	1.000000e+00	False	Stable_MCI (0.374) vs Fluctuating (0.405)
Stable_MCI	Dementia_to_MCI	1.196262	1.000000e+00	1.000000e+00	False	Stable_MCI (0.374) vs Dementia_to_MCI (0.333)
Stable_MCI	MCI_to_CN	0.996885	1.000000e+00	1.000000e+00	False	Stable_MCI (0.374) vs MCI_to_CN (0.375)
Stable_MCI	CN_to_Dementia	0.811749	5.777500e-01	1.000000e+00	False	Stable_MCI (0.374) vs CN_to_Dementia (0.424)
Stable_MCI	CN_to_MCI	1.072510	8.982989e-01	1.000000e+00	False	Stable_MCI (0.374) vs CN_to_MCI (0.358)
Stable_CN	Fluctuating	0.619302	6.656090e-02	1.000000e+00	False	Stable_CN (0.297) vs Fluctuating (0.405)
Stable_CN	Dementia_to_MCI	0.843305	1.000000e+00	1.000000e+00	False	Stable_CN (0.297) vs Dementia_to_MCI (0.333)
Stable_CN	MCI_to_CN	0.702754	2.246948e-01	1.000000e+00	False	Stable_CN (0.297) vs MCI_to_CN (0.375)
Stable_CN	CN_to_Dementia	0.572243	1.699282e-01	1.000000e+00	False	Stable_CN (0.297) vs CN_to_Dementia (0.424)
Stable_CN	CN_to_MCI	0.756066	2.980645e-01	1.000000e+00	False	Stable_CN (0.297) vs CN_to_MCI (0.358)
CN_to_MCI	CN_to_Dementia	0.756868	5.291144e-01	1.000000e+00	False	CN_to_MCI (0.358) vs CN_to_Dementia (0.424)
Dementia_to_MCI	Fluctuating	0.734375	1.000000e+00	1.000000e+00	False	Dementia_to_MCI (0.333) vs Fluctuating (0.405)

2.4.Baseline ADAS-cog13

--- ANOVA for ADAS13 ---

ANOVA p-value: 1.1294e-320

--- Tukey HSD Post Hoc for ADAS13 ---

Multiple Comparison of Means - Tukey HSD, FWER=0.05

group1	group2	meandiff	p-adj	lower	upper	reject
Fluctuating	Incline_CN_to_Dementia	-2.2657	0.502	-5.6832	1.1519	False
Fluctuating	Incline_CN_to_MCI	-1.4332	0.7374	-4.0291	1.1626	False
Fluctuating	Incline_MCI_to_Dementia	7.9364	0.0	5.869	10.0039	True
Fluctuating	Retrogression_Dementia_to_MCI	5.4731	0.7151	-4.2411	15.1873	False
Fluctuating	Retrogression_MCI_to_CN	-3.2009	0.0155	-6.0641	-0.3377	True
Fluctuating	Stable_CN	-4.3278	0.0	-6.3166	-2.339	True
Fluctuating	Stable_Dementia	16.7577	0.0	14.5138	19.0016	True
Fluctuating	Stable_MCI	1.4466	0.4105	-0.6021	3.4953	False
Incline_CN_to_Dementia	Incline_CN_to_MCI	0.8324	0.9978	-2.5729	4.2378	False
Incline_CN_to_Dementia	Incline_MCI_to_Dementia	10.2021	0.0	7.1802	13.224	True
Incline_CN_to_Dementia	Retrogression_Dementia_to_MCI	7.7388	0.2773	-2.2223	17.6999	False
Incline_CN_to_Dementia	Retrogression_MCI_to_CN	-0.9352	0.9968	-4.5485	2.678	False
Incline_CN_to_Dementia	Stable_CN	-2.0621	0.4343	-5.0308	0.9066	False
Incline_CN_to_Dementia	Stable_Dementia	19.0234	0.0	15.8781	22.1687	True
Incline_CN_to_Dementia	Stable_MCI	3.7123	0.0042	0.7031	6.7214	True
Incline_CN_to_MCI	Incline_MCI_to_Dementia	9.3697	0.0	7.3224	11.4169	True
Incline_CN_to_MCI	Retrogression_Dementia_to_MCI	6.9063	0.4	-2.8036	16.6162	False
Incline_CN_to_MCI	Retrogression_MCI_to_CN	-1.7677	0.5947	-4.6163	1.0809	False
Incline_CN_to_MCI	Stable_CN	-2.8946	0.0002	-4.8624	-0.9268	True
Incline_CN_to_MCI	Stable_Dementia	18.1909	0.0	15.9657	20.4162	True
Incline_CN_to_MCI	Stable_MCI	2.8798	0.0004	0.8515	4.9081	True
Incline_MCI_to_Dementia	Retrogression_Dementia_to_MCI	-2.4633	0.9969	-12.0455	7.1189	False
Incline_MCI_to_Dementia	Retrogression_MCI_to_CN	-11.1374	0.0	-13.5145	-8.7603	True
Incline_MCI_to_Dementia	Stable_CN	-12.2642	0.0	-13.4509	-11.0776	True
Incline_MCI_to_Dementia	Stable_Dementia	8.8213	0.0	7.244	10.3985	True
Incline_MCI_to_Dementia	Stable_MCI	-6.4898	0.0	-7.7743	-5.2054	True
Retrogression_Dementia_to_MCI	Retrogression_MCI_to_CN	-8.674	0.1304	-18.4588	1.1107	False
Retrogression_Dementia_to_MCI	Stable_CN	-9.8009	0.0397	-19.3664	-0.2354	True
Retrogression_Dementia_to_MCI	Stable_Dementia	11.2846	0.0085	1.6628	20.9064	True
Retrogression_Dementia_to_MCI	Stable_MCI	-4.0265	0.9298	-13.6047	5.5516	False
Retrogression_MCI_to_CN	Stable_CN	-1.1269	0.8483	-3.4359	1.1822	False
Retrogression_MCI_to_CN	Stable_Dementia	19.9586	0.0	17.4266	22.4907	True
Retrogression_MCI_to_CN	Stable_MCI	4.6475	0.0	2.2867	7.0083	True
Stable_CN	Stable_Dementia	21.0855	0.0	19.6128	22.5582	True
Stable_CN	Stable_MCI	5.7744	0.0	4.6207	6.9281	True
Stable_Dementia	Stable_MCI	-15.3111	0.0	-16.8637	-13.7585	True

2.5.Baseline MMSE

```
--- ANOVA for MMSE ---
ANOVA p-value: 8.8538e-293

--- Tukey HSD Post Hoc for MMSE ---
```

Multiple Comparison of Means - Tukey HSD, FWER=0.05

group1	group2	meandiff	p-adj	lower	upper	reject
Fluctuating	Incline_CN_to_Dementia	1.2295	0.0036	0.2422	2.2169	True
Fluctuating	Incline_CN_to_MCI	0.6652	0.1298	-0.0847	1.4152	False
Fluctuating	Incline_MCI_to_Dementia	-1.2155	0.0	-1.8128	-0.6182	True
Fluctuating	Retrogression_Dementia_to_MCI	-2.8917	0.0376	-5.6981	-0.0853	True
Fluctuating	Retrogression_MCI_to_CN	0.6873	0.1954	-0.1399	1.5144	False
Fluctuating	Stable_CN	0.9007	0.0	0.3262	1.4753	True
Fluctuating	Stable_Dementia	-5.1952	0.0	-5.8435	-4.547	True
Fluctuating	Stable_MCI	-0.2567	0.9168	-0.8486	0.3352	False
Incline_CN_to_Dementia	Incline_CN_to_MCI	-0.5643	0.6945	-1.5481	0.4195	False
Incline_CN_to_Dementia	Incline_MCI_to_Dementia	-2.4451	0.0	-3.3181	-1.572	True
Incline_CN_to_Dementia	Retrogression_Dementia_to_MCI	-4.1212	0.0003	-6.9989	-1.2435	True
Incline_CN_to_Dementia	Retrogression_MCI_to_CN	-0.5423	0.7976	-1.5861	0.5016	False
Incline_CN_to_Dementia	Stable_CN	-0.3288	0.9585	-1.1864	0.5289	False
Incline_CN_to_Dementia	Stable_Dementia	-6.4248	0.0	-7.3334	-5.5161	True
Incline_CN_to_Dementia	Stable_MCI	-1.4862	0.0	-2.3556	-0.6169	True
Incline_CN_to_MCI	Incline_MCI_to_Dementia	-1.8808	0.0	-2.4722	-1.2893	True
Incline_CN_to_MCI	Retrogression_Dementia_to_MCI	-3.5569	0.0028	-6.3621	-0.7517	True
Incline_CN_to_MCI	Retrogression_MCI_to_CN	0.022	1.0	-0.8009	0.845	False
Incline_CN_to_MCI	Stable_CN	0.2355	0.9353	-0.333	0.804	False
Incline_CN_to_MCI	Stable_Dementia	-5.8605	0.0	-6.5034	-5.2176	True
Incline_CN_to_MCI	Stable_MCI	-0.9219	0.0	-1.5079	-0.336	True
Incline_MCI_to_Dementia	Retrogression_Dementia_to_MCI	-1.6762	0.6273	-4.4444	1.0921	False
Incline_MCI_to_Dementia	Retrogression_MCI_to_CN	1.9028	0.0	1.2161	2.5895	True
Incline_MCI_to_Dementia	Stable_CN	2.1163	0.0	1.7734	2.4591	True
Incline_MCI_to_Dementia	Stable_Dementia	-3.9797	0.0	-4.4354	-3.5241	True
Incline_MCI_to_Dementia	Stable_MCI	0.9588	0.0	0.5877	1.3299	True
Retrogression_Dementia_to_MCI	Retrogression_MCI_to_CN	3.5789	0.0028	0.7522	6.4057	True
Retrogression_Dementia_to_MCI	Stable_CN	3.7924	0.0007	1.029	6.5559	True
Retrogression_Dementia_to_MCI	Stable_Dementia	-2.3036	0.1984	-5.0833	0.4761	False
Retrogression_Dementia_to_MCI	Stable_MCI	2.635	0.0764	-0.1321	5.4021	False
Retrogression_MCI_to_CN	Stable_CN	0.2135	0.9865	-0.4536	0.8805	False
Retrogression_MCI_to_CN	Stable_Dementia	-5.8825	0.0	-6.614	-5.151	True
Retrogression_MCI_to_CN	Stable_MCI	-0.944	0.0006	-1.626	-0.2619	True
Stable_CN	Stable_Dementia	-6.096	0.0	-6.5214	-5.6705	True
Stable_CN	Stable_MCI	-1.1574	0.0	-1.4907	-0.8242	True
Stable_Dementia	Stable_MCI	4.9385	0.0	4.49	5.3871	True

2.6.Baseline CDR

```
--- ANOVA for CDGLOBAL ---
ANOVA p-value: 0.0000e+00

--- Tukey HSD Post Hoc for CDGLOBAL ---
```

Multiple Comparison of Means - Tukey HSD, FWER=0.05

group1	group2	meandiff	p-adj	lower	upper	reject
Fluctuating	Incline_CN_to_Dementia	-0.3562	0.0	-0.4258	-0.2867	True
Fluctuating	Incline_CN_to_MCI	-0.3502	0.0	-0.403	-0.2973	True
Fluctuating	Incline_MCI_to_Dementia	0.1422	0.0	0.1001	0.1843	True
Fluctuating	Retrogression_Dementia_to_MCI	0.4771	0.0	0.2793	0.6749	True
Fluctuating	Retrogression_MCI_to_CN	0.135	0.0	0.0767	0.1933	True
Fluctuating	Stable_CN	-0.3553	0.0	-0.3957	-0.3148	True
Fluctuating	Stable_Dementia	0.3938	0.0	0.3481	0.4394	True
Fluctuating	Stable_MCI	0.1452	0.0	0.1035	0.1869	True
Incline_CN_to_Dementia	Incline_CN_to_MCI	0.0061	1.0	-0.0632	0.0754	False
Incline_CN_to_Dementia	Incline_MCI_to_Dementia	0.4984	0.0	0.4369	0.56	True
Incline_CN_to_Dementia	Retrogression_Dementia_to_MCI	0.8333	0.0	0.6305	1.0362	True
Incline_CN_to_Dementia	Retrogression_MCI_to_CN	0.4912	0.0	0.4177	0.5648	True
Incline_CN_to_Dementia	Stable_CN	0.001	1.0	-0.0595	0.0614	False
Incline_CN_to_Dementia	Stable_Dementia	0.75	0.0	0.686	0.814	True
Incline_CN_to_Dementia	Stable_MCI	0.5014	0.0	0.4402	0.5627	True
Incline_CN_to_MCI	Incline_MCI_to_Dementia	0.4923	0.0	0.4506	0.534	True
Incline_CN_to_MCI	Retrogression_Dementia_to_MCI	0.8272	0.0	0.6295	1.0249	True
Incline_CN_to_MCI	Retrogression_MCI_to_CN	0.4851	0.0	0.4271	0.5431	True
Incline_CN_to_MCI	Stable_CN	-0.0051	1.0	-0.0452	0.035	False
Incline_CN_to_MCI	Stable_Dementia	0.7439	0.0	0.6986	0.7892	True
Incline_CN_to_MCI	Stable_MCI	0.4953	0.0	0.454	0.5366	True
Incline_MCI_to_Dementia	Retrogression_Dementia_to_MCI	0.3349	0.0	0.1398	0.53	True
Incline_MCI_to_Dementia	Retrogression_MCI_to_CN	-0.0072	0.9999	-0.0556	0.0412	False
Incline_MCI_to_Dementia	Stable_CN	-0.4974	0.0	-0.5216	-0.4733	True
Incline_MCI_to_Dementia	Stable_Dementia	0.2516	0.0	0.2195	0.2837	True
Incline_MCI_to_Dementia	Stable_MCI	0.003	1.0	-0.0231	0.0292	False
Retrogression_Dementia_to_MCI	Retrogression_MCI_to_CN	-0.3421	0.0	-0.5413	-0.1429	True
Retrogression_Dementia_to_MCI	Stable_CN	-0.8323	0.0	-1.0271	-0.6376	True
Retrogression_Dementia_to_MCI	Stable_Dementia	-0.0833	0.9252	-0.2793	0.1126	False
Retrogression_Dementia_to_MCI	Stable_MCI	-0.3319	0.0	-0.5269	-0.1369	True
Retrogression_MCI_to_CN	Stable_CN	-0.4902	0.0	-0.5372	-0.4432	True
Retrogression_MCI_to_CN	Stable_Dementia	0.2588	0.0	0.2072	0.3103	True
Retrogression_MCI_to_CN	Stable_MCI	0.0102	0.9992	-0.0379	0.0583	False
Stable_CN	Stable_Dementia	0.749	0.0	0.719	0.779	True
Stable_CN	Stable_MCI	0.5004	0.0	0.477	0.5239	True
Stable_Dementia	Stable_MCI	-0.2486	0.0	-0.2802	-0.2169	True

2.7.Baseline CDR-SB

```
--- ANOVA for CDRSB ---
ANOVA p-value: 0.0000e+00

--- Tukey HSD Post Hoc for CDRSB ---
```

Multiple Comparison of Means - Tukey HSD, FWER=0.05

group1	group2	meandiff	p-adj	lower	upper	reject
Fluctuating	Incline_CN_to_Dementia	-1.042	0.0	-1.5598	-0.5243	True
Fluctuating	Incline_CN_to_MCI	-1.0204	0.0	-1.4137	-0.6271	True
Fluctuating	Incline_MCI_to_Dementia	0.7653	0.0	0.4521	1.0786	True
Fluctuating	Retrogression_Dementia_to_MCI	2.4125	0.0	0.9407	3.8843	True
Fluctuating	Retrogression_MCI_to_CN	-0.0787	0.9998	-0.5125	0.3551	False
Fluctuating	Stable_CN	-1.0606	0.0	-1.3619	-0.7592	True
Fluctuating	Stable_Dementia	3.2339	0.0	2.894	3.5739	True
Fluctuating	Stable_MCI	0.133	0.9222	-0.1774	0.4434	False
Incline_CN_to_Dementia	Incline_CN_to_MCI	0.0216	1.0	-0.4943	0.5376	False
Incline_CN_to_Dementia	Incline_MCI_to_Dementia	1.8074	0.0	1.3495	2.2652	True
Incline_CN_to_Dementia	Retrogression_Dementia_to_MCI	3.4545	0.0	1.9453	4.9638	True
Incline_CN_to_Dementia	Retrogression_MCI_to_CN	0.9633	0.0	0.4159	1.5108	True
Incline_CN_to_Dementia	Stable_CN	-0.0185	1.0	-0.4683	0.4313	False
Incline_CN_to_Dementia	Stable_Dementia	4.276	0.0	3.7994	4.7525	True
Incline_CN_to_Dementia	Stable_MCI	1.175	0.0	0.7191	1.6309	True
Incline_CN_to_MCI	Incline_MCI_to_Dementia	1.7858	0.0	1.4756	2.096	True
Incline_CN_to_MCI	Retrogression_Dementia_to_MCI	3.4329	0.0	1.9618	4.9041	True
Incline_CN_to_MCI	Retrogression_MCI_to_CN	0.9417	0.0	0.5101	1.3733	True
Incline_CN_to_MCI	Stable_CN	-0.0401	1.0	-0.3383	0.258	False
Incline_CN_to_MCI	Stable_Dementia	4.2544	0.0	3.9172	4.5915	True
Incline_CN_to_MCI	Stable_MCI	1.1534	0.0	0.8461	1.4607	True
Incline_MCI_to_Dementia	Retrogression_Dementia_to_MCI	1.6472	0.013	0.1953	3.099	True
Incline_MCI_to_Dementia	Retrogression_MCI_to_CN	-0.8441	0.0	-1.2042	-0.4839	True
Incline_MCI_to_Dementia	Stable_CN	-1.8259	0.0	-2.0057	-1.6461	True
Incline_MCI_to_Dementia	Stable_Dementia	2.4686	0.0	2.2296	2.7075	True
Incline_MCI_to_Dementia	Stable_MCI	-0.6324	0.0	-0.827	-0.4378	True
Retrogression_Dementia_to_MCI	Retrogression_MCI_to_CN	-2.4912	0.0	-3.9737	-1.0087	True
Retrogression_Dementia_to_MCI	Stable_CN	-3.4731	0.0	-4.9223	-2.0238	True
Retrogression_Dementia_to_MCI	Stable_Dementia	0.8214	0.715	-0.6364	2.2792	False
Retrogression_Dementia_to_MCI	Stable_MCI	-2.2795	0.0	-3.7307	-0.8283	True
Retrogression_MCI_to_CN	Stable_CN	-0.9818	0.0	-1.3317	-0.632	True
Retrogression_MCI_to_CN	Stable_Dementia	3.3127	0.0	2.929	3.6963	True
Retrogression_MCI_to_CN	Stable_MCI	0.2117	0.6565	-0.146	0.5694	False
Stable_CN	Stable_Dementia	4.2945	0.0	4.0714	4.5176	True
Stable_CN	Stable_MCI	1.1935	0.0	1.0187	1.3683	True
Stable_Dementia	Stable_MCI	-3.101	0.0	-3.3362	-2.8657	True

2.8.A+

Chi-squared test p-value: 7.9653e-53

Post-hoc pairwise Fisher exact tests (Holm correction):

Group1	Group2	OddsRatio	p_raw	p_adj_holm	significant_holm_0.05	A+_prop_direction
Stable_CN	MCI_to_Dementia	0.071459	1.970223e-46	7.092803e-45	True	Stable_CN (0.348) vs MCI_to_Dementia (0.882)
Stable_CN	Stable_Dementia	0.060844	2.464687e-30	8.626406e-29	True	Stable_CN (0.348) vs Stable_Dementia (0.898)
Stable_MCI	MCI_to_Dementia	0.122701	1.684198e-25	5.726274e-24	True	Stable_MCI (0.478) vs MCI_to_Dementia (0.882)
Stable_MCI	Stable_Dementia	0.104475	8.022015e-18	2.647265e-16	True	Stable_MCI (0.478) vs Stable_Dementia (0.898)
CN_to_MCI	MCI_to_Dementia	0.127060	4.100437e-12	1.312140e-10	True	CN_to_MCI (0.487) vs MCI_to_Dementia (0.882)
MCI_to_Dementia	MCI_to_CN	10.181818	7.791731e-12	2.415437e-10	True	MCI_to_Dementia (0.882) vs MCI_to_CN (0.423)
Stable_Dementia	MCI_to_CN	11.958042	1.106861e-10	3.320584e-09	True	Stable_Dementia (0.898) vs MCI_to_CN (0.423)
Stable_Dementia	CN_to_MCI	9.243243	1.609957e-10	4.668874e-09	True	Stable_Dementia (0.898) vs CN_to_MCI (0.487)
MCI_to_Dementia	Fluctuating	6.191870	1.877363e-09	5.256616e-08	True	MCI_to_Dementia (0.882) vs Fluctuating (0.547)
Stable_Dementia	Fluctuating	7.272045	2.720752e-08	7.346031e-07	True	Stable_Dementia (0.898) vs Fluctuating (0.547)
Stable_CN	CN_to_Dementia	0.203260	8.987558e-05	2.336765e-03	True	Stable_CN (0.348) vs CN_to_Dementia (0.724)
Stable_CN	Stable_MCI	0.582379	3.509929e-04	8.774822e-03	True	Stable_CN (0.348) vs Stable_MCI (0.478)
Stable_CN	Fluctuating	0.442462	1.261934e-03	3.028642e-02	True	Stable_CN (0.348) vs Fluctuating (0.547)
CN_to_Dementia	MCI_to_CN	3.579545	1.128806e-02	2.596253e-01	False	CN_to_Dementia (0.724) vs MCI_to_CN (0.423)
Stable_MCI	CN_to_Dementia	0.349016	1.193698e-02	2.626135e-01	False	Stable_MCI (0.478) vs CN_to_Dementia (0.724)
Stable_Dementia	CN_to_Dementia	3.340659	2.941902e-02	5.963852e-01	False	Stable_Dementia (0.898) vs CN_to_Dementia (0.724)
Stable_CN	CN_to_MCI	0.562398	2.839930e-02	5.963852e-01	False	Stable_CN (0.348) vs CN_to_MCI (0.487)
CN_to_Dementia	MCI_to_Dementia	0.351562	3.771347e-02	7.165559e-01	False	CN_to_Dementia (0.724) vs MCI_to_Dementia (0.882)
CN_to_MCI	CN_to_Dementia	0.361416	4.716858e-02	8.490344e-01	False	CN_to_MCI (0.487) vs CN_to_Dementia (0.724)
MCI_to_Dementia	Dementia_to_MCI	7.466667	2.279412e-01	1.000000e+00	False	MCI_to_Dementia (0.882) vs Dementia_to_MCI (0.500)
CN_to_Dementia	Fluctuating	2.176829	1.211394e-01	1.000000e+00	False	CN_to_Dementia (0.724) vs Fluctuating (0.547)
CN_to_Dementia	Dementia_to_MCI	2.625000	5.032258e-01	1.000000e+00	False	CN_to_Dementia (0.724) vs Dementia_to_MCI (0.500)
MCI_to_CN	Dementia_to_MCI	0.733333	1.000000e+00	1.000000e+00	False	MCI_to_CN (0.423) vs Dementia_to_MCI (0.500)
CN_to_MCI	Fluctuating	0.786742	5.162304e-01	1.000000e+00	False	CN_to_MCI (0.487) vs Fluctuating (0.547)
Stable_Dementia	MCI_to_Dementia	1.174451	7.327562e-01	1.000000e+00	False	Stable_Dementia (0.898) vs MCI_to_Dementia (0.882)
CN_to_MCI	MCI_to_CN	1.293706	5.883727e-01	1.000000e+00	False	CN_to_MCI (0.487) vs MCI_to_CN (0.423)
Stable_Dementia	Dementia_to_MCI	8.769231	2.060320e-01	1.000000e+00	False	Stable_Dementia (0.898) vs Dementia_to_MCI (0.500)
MCI_to_CN	Fluctuating	0.608130	2.075681e-01	1.000000e+00	False	MCI_to_CN (0.423) vs Fluctuating (0.547)
Stable_MCI	Fluctuating	0.759749	3.064030e-01	1.000000e+00	False	Stable_MCI (0.478) vs Fluctuating (0.547)
Stable_MCI	Dementia_to_MCI	0.916168	1.000000e+00	1.000000e+00	False	Stable_MCI (0.478) vs Dementia_to_MCI (0.500)
Stable_MCI	MCI_to_CN	1.249320	5.494649e-01	1.000000e+00	False	Stable_MCI (0.478) vs MCI_to_CN (0.423)
Stable_MCI	CN_to_MCI	0.965690	8.991210e-01	1.000000e+00	False	Stable_MCI (0.478) vs CN_to_MCI (0.487)
Stable_CN	Dementia_to_MCI	0.533557	1.000000e+00	1.000000e+00	False	Stable_CN (0.348) vs Dementia_to_MCI (0.500)
Stable_CN	MCI_to_CN	0.727578	2.882155e-01	1.000000e+00	False	Stable_CN (0.348) vs MCI_to_CN (0.423)
CN_to_MCI	Dementia_to_MCI	0.948718	1.000000e+00	1.000000e+00	False	CN_to_MCI (0.487) vs Dementia_to_MCI (0.500)
Dementia_to_MCI	Fluctuating	0.829268	1.000000e+00	1.000000e+00	False	Dementia_to_MCI (0.500) vs Fluctuating (0.547)

2.9.T+

Chi-squared test p-value: 5.4440e-43

Post-hoc pairwise Fisher exact tests (Holm correction):

Group1	Group2	OddsRatio	p_raw	p_adj_holm	significant_holm_0.05	T+_prop_direction
Stable_CN	MCI_to_Dementia	0.140484	1.006336e-29	3.622810e-28	True	Stable_CN (0.303) vs MCI_to_Dementia (0.755)
Stable_CN	Stable_Dementia	0.072971	2.736176e-29	9.576617e-28	True	Stable_CN (0.303) vs Stable_Dementia (0.856)
Stable_MCI	Stable_Dementia	0.124939	5.713760e-17	1.942678e-15	True	Stable_MCI (0.426) vs Stable_Dementia (0.856)
Stable_Dementia	MCI_to_CN	19.994949	9.163439e-15	3.023935e-13	True	Stable_Dementia (0.856) vs MCI_to_CN (0.229)
Stable_MCI	MCI_to_Dementia	0.240530	1.739054e-14	5.564973e-13	True	Stable_MCI (0.426) vs MCI_to_Dementia (0.755)
Stable_Dementia	Fluctuating	11.641204	2.964976e-13	9.191427e-12	True	Stable_Dementia (0.856) vs Fluctuating (0.338)
MCI_to_Dementia	MCI_to_CN	10.385965	9.943862e-12	2.983159e-10	True	MCI_to_Dementia (0.755) vs MCI_to_CN (0.229)
MCI_to_Dementia	Fluctuating	6.046784	2.587829e-10	7.504705e-09	True	MCI_to_Dementia (0.755) vs Fluctuating (0.338)
Stable_Dementia	CN_to_MCI	6.119281	1.055949e-07	2.956658e-06	True	Stable_Dementia (0.856) vs CN_to_MCI (0.493)
CN_to_MCI	MCI_to_Dementia	0.314610	8.424340e-05	2.274572e-03	True	CN_to_MCI (0.493) vs MCI_to_Dementia (0.755)
Stable_CN	Stable_MCI	0.584059	6.994301e-04	1.818518e-02	True	Stable_CN (0.303) vs Stable_MCI (0.426)
Stable_Dementia	CN_to_Dementia	4.670635	1.645141e-03	4.112853e-02	True	Stable_Dementia (0.856) vs CN_to_Dementia (0.560)
Stable_CN	CN_to_MCI	0.446533	2.358716e-03	5.660918e-02	False	Stable_CN (0.303) vs CN_to_MCI (0.493)
CN_to_MCI	MCI_to_CN	3.267532	6.499124e-03	1.494798e-01	False	CN_to_MCI (0.493) vs MCI_to_CN (0.229)
CN_to_Dementia	MCI_to_CN	4.280992	8.575552e-03	1.886622e-01	False	CN_to_Dementia (0.560) vs MCI_to_CN (0.229)
Stable_MCI	MCI_to_CN	2.498139	1.072075e-02	2.251357e-01	False	Stable_MCI (0.426) vs MCI_to_CN (0.229)
Stable_CN	CN_to_Dementia	0.340823	1.309573e-02	2.619146e-01	False	Stable_CN (0.303) vs CN_to_Dementia (0.560)
Stable_Dementia	MCI_to_Dementia	1.925189	2.916052e-02	5.540499e-01	False	Stable_Dementia (0.856) vs MCI_to_Dementia (0.755)
CN_to_Dementia	MCI_to_Dementia	0.412190	5.325243e-02	9.585437e-01	False	CN_to_Dementia (0.560) vs MCI_to_Dementia (0.755)
MCI_to_CN	Dementia_to_MCI	0.000000	2.448980e-01	1.000000e+00	False	MCI_to_CN (0.229) vs Dementia_to_MCI (1.000)
Stable_CN	MCI_to_CN	1.459061	3.217279e-01	1.000000e+00	False	Stable_CN (0.303) vs MCI_to_CN (0.229)
MCI_to_Dementia	Dementia_to_MCI	0.000000	1.000000e+00	1.000000e+00	False	MCI_to_Dementia (0.755) vs Dementia_to_MCI (1.000)
Stable_CN	Dementia_to_MCI	0.000000	3.041475e-01	1.000000e+00	False	Stable_CN (0.303) vs Dementia_to_MCI (1.000)
CN_to_Dementia	Fluctuating	2.492424	6.040372e-02	1.000000e+00	False	CN_to_Dementia (0.560) vs Fluctuating (0.338)
CN_to_Dementia	Dementia_to_MCI	0.000000	1.000000e+00	1.000000e+00	False	CN_to_Dementia (0.560) vs Dementia_to_MCI (1.000)
Stable_CN	Fluctuating	0.849476	5.796713e-01	1.000000e+00	False	Stable_CN (0.303) vs Fluctuating (0.338)
Stable_MCI	Fluctuating	1.454435	1.824211e-01	1.000000e+00	False	Stable_MCI (0.426) vs Fluctuating (0.338)
CN_to_MCI	Fluctuating	1.902381	8.591974e-02	1.000000e+00	False	CN_to_MCI (0.493) vs Fluctuating (0.338)
CN_to_MCI	Dementia_to_MCI	0.000000	1.000000e+00	1.000000e+00	False	CN_to_MCI (0.493) vs Dementia_to_MCI (1.000)
Stable_MCI	CN_to_MCI	0.764534	3.471011e-01	1.000000e+00	False	Stable_MCI (0.426) vs CN_to_MCI (0.493)
Stable_MCI	CN_to_Dementia	0.583542	2.130392e-01	1.000000e+00	False	Stable_MCI (0.426) vs CN_to_Dementia (0.560)
CN_to_MCI	CN_to_Dementia	0.763265	6.436832e-01	1.000000e+00	False	CN_to_MCI (0.493) vs CN_to_Dementia (0.560)
Stable_MCI	Dementia_to_MCI	0.000000	4.280936e-01	1.000000e+00	False	Stable_MCI (0.426) vs Dementia_to_MCI (1.000)
Stable_Dementia	Dementia_to_MCI	0.000000	1.000000e+00	1.000000e+00	False	Stable_Dementia (0.856) vs Dementia_to_MCI (1.000)
MCI_to_CN	Fluctuating	0.582207	2.245090e-01	1.000000e+00	False	MCI_to_CN (0.229) vs Fluctuating (0.338)
Dementia_to_MCI	Fluctuating	inf	3.472222e-01	1.000000e+00	False	Dementia_to_MCI (1.000) vs Fluctuating (0.338)

2.10. Cardiovascular disease history

Chi-squared test p-value: 1.5782e-01

Post-hoc pairwise Fisher exact tests (Holm correction):

Group1	Group2	OddsRatio	p_raw	p_adj_holm	significant_holm_0.05	CVDYes_prop_direction
Stable_CN	Stable_MCI	0.688647	0.036283	1.0	False	Stable_CN (0.653) vs Stable_MCI (0.732)
Stable_Dementia	Fluctuating	1.007080	1.000000	1.0	False	Stable_Dementia (0.655) vs Fluctuating (0.653)
CN_to_MCI	CN_to_Dementia	1.408696	0.615829	1.0	False	CN_to_MCI (0.783) vs CN_to_Dementia (0.719)
CN_to_MCI	MCI_to_Dementia	1.369014	0.365464	1.0	False	CN_to_MCI (0.783) vs MCI_to_Dementia (0.724)
CN_to_MCI	MCI_to_CN	2.110345	0.090917	1.0	False	CN_to_MCI (0.783) vs MCI_to_CN (0.630)
CN_to_MCI	Dementia_to_MCI	0.000000	1.000000	1.0	False	CN_to_MCI (0.783) vs Dementia_to_MCI (1.000)
CN_to_MCI	Fluctuating	1.910204	0.098475	1.0	False	CN_to_MCI (0.783) vs Fluctuating (0.653)
Stable_Dementia	Dementia_to_MCI	0.000000	0.547883	1.0	False	Stable_Dementia (0.655) vs Dementia_to_MCI (1.000)
CN_to_Dementia	MCI_to_Dementia	0.971831	1.000000	1.0	False	CN_to_Dementia (0.719) vs MCI_to_Dementia (0.724)
CN_to_Dementia	Dementia_to_MCI	0.000000	1.000000	1.0	False	CN_to_Dementia (0.719) vs Dementia_to_MCI (1.000)
CN_to_Dementia	Fluctuating	1.356009	0.653392	1.0	False	CN_to_Dementia (0.719) vs Fluctuating (0.653)
MCI_to_Dementia	MCI_to_CN	1.541507	0.220494	1.0	False	MCI_to_Dementia (0.724) vs MCI_to_CN (0.630)
MCI_to_Dementia	Dementia_to_MCI	0.000000	1.000000	1.0	False	MCI_to_Dementia (0.724) vs Dementia_to_MCI (1.000)
MCI_to_Dementia	Fluctuating	1.395314	0.254360	1.0	False	MCI_to_Dementia (0.724) vs Fluctuating (0.653)
MCI_to_CN	Dementia_to_MCI	0.000000	0.532801	1.0	False	MCI_to_CN (0.630) vs Dementia_to_MCI (1.000)
CN_to_Dementia	MCI_to_CN	1.498084	0.471025	1.0	False	CN_to_Dementia (0.719) vs MCI_to_CN (0.630)
Stable_Dementia	MCI_to_CN	1.112597	0.859134	1.0	False	Stable_Dementia (0.655) vs MCI_to_CN (0.630)
Stable_Dementia	MCI_to_Dementia	0.721759	0.147256	1.0	False	Stable_Dementia (0.655) vs MCI_to_Dementia (0.724)
Stable_Dementia	CN_to_Dementia	0.742680	0.540084	1.0	False	Stable_Dementia (0.655) vs CN_to_Dementia (0.719)
Stable_CN	Stable_Dementia	0.992057	1.000000	1.0	False	Stable_CN (0.653) vs Stable_Dementia (0.655)
Stable_CN	CN_to_MCI	0.523023	0.046461	1.0	False	Stable_CN (0.653) vs CN_to_MCI (0.783)
Stable_CN	CN_to_Dementia	0.736780	0.558975	1.0	False	Stable_CN (0.653) vs CN_to_Dementia (0.719)
Stable_CN	MCI_to_Dementia	0.716026	0.067163	1.0	False	Stable_CN (0.653) vs MCI_to_Dementia (0.724)
Stable_CN	MCI_to_CN	1.103759	0.744207	1.0	False	Stable_CN (0.653) vs MCI_to_CN (0.630)
Stable_CN	Dementia_to_MCI	0.000000	0.546816	1.0	False	Stable_CN (0.653) vs Dementia_to_MCI (1.000)
Stable_CN	Fluctuating	0.999081	1.000000	1.0	False	Stable_CN (0.653) vs Fluctuating (0.653)
Stable_MCI	Stable_Dementia	1.440588	0.115949	1.0	False	Stable_MCI (0.732) vs Stable_Dementia (0.655)
Stable_MCI	CN_to_MCI	0.759494	0.446778	1.0	False	Stable_MCI (0.732) vs CN_to_MCI (0.783)
Stable_MCI	CN_to_Dementia	1.069895	0.836432	1.0	False	Stable_MCI (0.732) vs CN_to_Dementia (0.719)
Stable_MCI	MCI_to_Dementia	1.039758	0.853389	1.0	False	Stable_MCI (0.732) vs MCI_to_Dementia (0.724)
Stable_MCI	MCI_to_CN	1.602794	0.161375	1.0	False	Stable_MCI (0.732) vs MCI_to_CN (0.630)
Stable_MCI	Dementia_to_MCI	0.000000	1.000000	1.0	False	Stable_MCI (0.732) vs Dementia_to_MCI (1.000)
Stable_MCI	Fluctuating	1.450788	0.197185	1.0	False	Stable_MCI (0.732) vs Fluctuating (0.653)
Stable_Dementia	CN_to_MCI	0.527211	0.078569	1.0	False	Stable_Dementia (0.655) vs CN_to_MCI (0.783)
MCI_to_CN	Fluctuating	0.905162	0.846189	1.0	False	MCI_to_CN (0.630) vs Fluctuating (0.653)
Dementia_to_MCI	Fluctuating	inf	0.546822	1.0	False	Dementia_to_MCI (1.000) vs Fluctuating (0.653)

2.11. Psychiatric symptom history

Chi-squared test p-value: 2.9199e-04

Post-hoc pairwise Fisher exact tests (Holm correction):

Group1	Group2	OddsRatio	p_raw	p_adj_holm	significant_holm_0.05	PsychYes_prop_direction
Stable_CN	MCI_to_Dementia	0.454180	0.000006	0.000218	True	Stable_CN (0.259) vs MCI_to_Dementia (0.435)
Stable_CN	Stable_Dementia	0.451885	0.000211	0.007383	True	Stable_CN (0.259) vs Stable_Dementia (0.437)
Stable_CN	Stable_MCI	0.556037	0.000989	0.033612	True	Stable_CN (0.259) vs Stable_MCI (0.386)
Stable_CN	Fluctuating	0.470596	0.006905	0.227852	False	Stable_CN (0.259) vs Fluctuating (0.427)
CN_to_MCI	MCI_to_Dementia	0.492812	0.014656	0.468990	False	CN_to_MCI (0.275) vs MCI_to_Dementia (0.435)
Stable_Dementia	CN_to_MCI	2.039474	0.024711	0.766032	False	Stable_Dementia (0.437) vs CN_to_MCI (0.275)
CN_to_MCI	MCI_to_CN	0.591111	0.224295	1.000000	False	CN_to_MCI (0.275) vs MCI_to_CN (0.391)
CN_to_MCI	Dementia_to_MCI	0.380000	0.486922	1.000000	False	CN_to_MCI (0.275) vs Dementia_to_MCI (0.500)
CN_to_MCI	Fluctuating	0.510625	0.080753	1.000000	False	CN_to_MCI (0.275) vs Fluctuating (0.427)
CN_to_Dementia	MCI_to_Dementia	0.778125	0.575788	1.000000	False	CN_to_Dementia (0.375) vs MCI_to_Dementia (0.435)
CN_to_Dementia	Dementia_to_MCI	0.600000	1.000000	1.000000	False	CN_to_Dementia (0.375) vs Dementia_to_MCI (0.500)
CN_to_MCI	CN_to_Dementia	0.633333	0.357455	1.000000	False	CN_to_MCI (0.275) vs CN_to_Dementia (0.375)
CN_to_Dementia	Fluctuating	0.806250	0.672198	1.000000	False	CN_to_Dementia (0.375) vs Fluctuating (0.427)
MCI_to_Dementia	MCI_to_CN	1.199465	0.632740	1.000000	False	MCI_to_Dementia (0.435) vs MCI_to_CN (0.391)
MCI_to_Dementia	Dementia_to_MCI	0.771084	1.000000	1.000000	False	MCI_to_Dementia (0.435) vs Dementia_to_MCI (0.500)
MCI_to_Dementia	Fluctuating	1.036145	1.000000	1.000000	False	MCI_to_Dementia (0.435) vs Fluctuating (0.427)
MCI_to_CN	Dementia_to_MCI	0.642857	1.000000	1.000000	False	MCI_to_CN (0.391) vs Dementia_to_MCI (0.500)
CN_to_Dementia	MCI_to_CN	0.933333	1.000000	1.000000	False	CN_to_Dementia (0.375) vs MCI_to_CN (0.391)
Stable_Dementia	Fluctuating	1.041406	1.000000	1.000000	False	Stable_Dementia (0.437) vs Fluctuating (0.427)
Stable_Dementia	MCI_to_Dementia	1.005078	1.000000	1.000000	False	Stable_Dementia (0.437) vs MCI_to_Dementia (0.435)
Stable_Dementia	MCI_to_CN	1.205556	0.611487	1.000000	False	Stable_Dementia (0.437) vs MCI_to_CN (0.391)
MCI_to_CN	Fluctuating	0.863839	0.849325	1.000000	False	MCI_to_CN (0.391) vs Fluctuating (0.427)
Stable_Dementia	CN_to_Dementia	1.291667	0.558887	1.000000	False	Stable_Dementia (0.437) vs CN_to_Dementia (0.375)
Stable_MCI	Fluctuating	0.846340	0.596883	1.000000	False	Stable_MCI (0.386) vs Fluctuating (0.427)
Stable_MCI	Dementia_to_MCI	0.629834	1.000000	1.000000	False	Stable_MCI (0.386) vs Dementia_to_MCI (0.500)
Stable_MCI	MCI_to_CN	0.979742	1.000000	1.000000	False	Stable_MCI (0.386) vs MCI_to_CN (0.391)
Stable_MCI	MCI_to_Dementia	0.816816	0.241540	1.000000	False	Stable_MCI (0.386) vs MCI_to_Dementia (0.435)
Stable_MCI	CN_to_Dementia	1.049724	1.000000	1.000000	False	Stable_MCI (0.386) vs CN_to_Dementia (0.375)
Stable_MCI	CN_to_MCI	1.657459	0.096186	1.000000	False	Stable_MCI (0.386) vs CN_to_MCI (0.275)
Stable_MCI	Stable_Dementia	0.812689	0.348988	1.000000	False	Stable_MCI (0.386) vs Stable_Dementia (0.437)
Stable_CN	Dementia_to_MCI	0.350211	0.454287	1.000000	False	Stable_CN (0.259) vs Dementia_to_MCI (0.500)
Stable_CN	MCI_to_CN	0.544773	0.076935	1.000000	False	Stable_CN (0.259) vs MCI_to_CN (0.391)
Stable_CN	CN_to_Dementia	0.583685	0.208473	1.000000	False	Stable_CN (0.259) vs CN_to_Dementia (0.375)
Stable_CN	CN_to_MCI	0.921608	0.764925	1.000000	False	Stable_CN (0.259) vs CN_to_MCI (0.275)
Stable_Dementia	Dementia_to_MCI	0.775000	1.000000	1.000000	False	Stable_Dementia (0.437) vs Dementia_to_MCI (0.500)
Dementia_to_MCI	Fluctuating	1.343750	1.000000	1.000000	False	Dementia_to_MCI (0.500) vs Fluctuating (0.427)

Supplementary Table 3. Effect sizes for trajectory subclass comparisons

=================== SUPPLEMENT: EFFECT SIZES (SUBCLASSES) ===================

Measure	N_CN	N_MCI	N_Dementia	N_CN→MCI	N_CN→Dementia	N_MCI→Dementia	N_MCI→CN	N_Dementia→MCI	N_Fluctuation	N_total	effect_name	effect
Age (baseline)	501	347	168	82	33	316	57	3	80	1587	η^2	0.047764
Follow-up duration (years)	501	347	168	82	33	316	57	3	80	1587	η^2	0.148055
ADAS-Cog 13 (baseline)	501	347	168	82	33	316	57	3	80	1587	η^2	0.615180
MMSE (baseline)	501	347	168	82	33	316	57	3	80	1587	η^2	0.582456
CDR global (baseline)	501	347	168	82	33	316	57	3	80	1587	η^2	0.863940
CDR-SB (baseline)	501	347	168	82	33	316	57	3	80	1587	η^2	0.719603
Sex	501	347	168	82	33	316	57	3	80	1587	Cramér's V	0.170187
APOE ε4 carrier	501	347	168	82	33	316	57	3	80	1587	Cramér's V	0.287733
A status	455	319	127	76	28	254	52	2	75	1388	Cramér's V	0.436165
T status	415	289	124	62	24	230	46	1	67	1258	Cramér's V	0.414506
CVD history	320	291	138	69	32	294	46	2	75	1267	Cramér's V	0.095588
Psych history	320	291	138	69	32	294	46	2	75	1267	Cramér's V	0.154253

References

1. Bittner T, Zetterberg H, Teunissen CE, et al. Technical performance of a novel, fully automated electrochemiluminescence immunoassay for the quantitation of β-amyloid (1-42) in human cerebrospinal fluid. *Alzheimers Dement J Alzheimers Assoc*. 2016;12(5):517-526. doi:10.1016/j.jalz.2015.09.009

2. Shaw LM, Hansson O, Manuilova E, et al. Method comparison study of the Elecsys® β-Amyloid (1-42) CSF assay versus comparator assays and LC-MS/MS. *Clin Biochem*. 2019;72:7-14. doi:10.1016/j.clinbiochem.2019.05.006

3. Leinenbach A, Pannee J, Dülffer T, et al. Mass spectrometry-based candidate reference measurement procedure for quantification of amyloid-β in cerebrospinal fluid. *Clin Chem*. 2014;60(7):987-994. doi:10.1373/clinchem.2013.220392

4. Royse SK, Minhas DS, Lopresti BJ, et al. Validation of amyloid PET positivity thresholds in centiloids: a multisite PET study approach. *Alzheimers Res Ther*. 2021;13(1):99. doi:10.1186/s13195-021-00836-1

5. Guo T, Korman D, La Joie R, et al. Normalization of CSF pTau measurement by Aβ40 improves its performance as a biomarker of Alzheimer's disease. *Alzheimers Res Ther*. 2020;12(1):97. doi:10.1186/s13195-020-00665-8

6. Jagust WJ, Koeppe RA, Rabinovici GD, et al. The ADNI PET Core at 20. *Alzheimers Dement*. 2024;20(10):7340-7349. doi:10.1002/alz.14165

7. Klunk WE, Koeppe RA, Price JC, et al. The Centiloid Project: standardizing quantitative amyloid plaque estimation by PET. *Alzheimers Dement J Alzheimers Assoc*. 2015;11(1):1-15.e1-4. doi:10.1016/j.jalz.2014.07.003
